# Response conflicts in visually guided movements under mental fatigue

**DOI:** 10.1007/s00426-026-02293-7

**Published:** 2026-04-24

**Authors:** Árpád Csathó, Dimitri Van der Linden, Gergő Jakóczi, Rebeka Gőgös, András Matuz

**Affiliations:** 1 Department of Behavioural Sciences, Medical School, University of Pécs, Szigeti str. 12, Pécs, H-7624 Hungary; 2 Szentágothai Research Centre, University of Pécs, Pécs, Hungary; 3 Department of Psychology, Education, and Child Studies, Erasmus University Rotterdam, Rotterdam, the Netherlands; 4 Institute of Biology, University of Pécs, Pécs, Hungary

**Keywords:** Fatigue, Time-on-task, Visually guided movement, Response conflict, Cognitive control

## Abstract

Mental fatigue induced by prolonged task performance (time-on-task [ToT]) is typically perceived as an unpleasant state and is associated with compromised performance. Operations requiring cognitive control, such as resolving response conflicts, are particularly sensitive to ToT-related detrimental effects. As cognitive control is critical for precise movements, ToT likely impacts movement performance. Two experiments explored this by examining the resolution of response conflicts in pointing movement with increasing ToT. In Experiment 1, participants performed a flanker task with congruent, neutral, and incongruent pointing trials. The results revealed slower movement initiation and execution, especially in incongruent trials, and ToT-related impairments in leftward movements possibly due to rightward attentional bias under fatigue. Experiment 2 introduced ‘leap trials,’ where spatial target locations shifted unexpectedly. While leap trials required high cognitive control, they resisted ToT-related deterioration, suggesting that phasic changes in spatial orientation resist fatigue. The performance of frequent normal trials with stable target locations declined over time, indicating that reduced tonic vigilance may worsen movements. Across both experiments, motivation manipulations after ToT periods mitigated declines in performance, reflecting the compensatory role of increased task engagement. These findings highlight the interplay between fatigue and cognitive control in motor tasks, offering insights into mechanisms underlying performance under mental strain.

**Supplementary Information:**

The online version contains supplementary material available at 10.1007/s00426-026-02293-7.

## Introduction

Mental fatigue (sometimes also labelled ‘cognitive fatigue’) is a highly complex biopsychological state that involves changes in mood, motivation, and behaviour and typically occurs while, or after performing a demanding mental task for a prolonged period of time. It is a powerful behavioural determinant, playing a decisive role in many voluntary and involuntary actions both in laboratory experiments and in everyday situations (Bener et al., 2017; Lal & Craig, 2001). Fatigue is usually perceived as an unpleasant feeling that is accompanied by an increasing urge to stop putting effort into a task. In addition, it tends to coincide with compromised performance, even though it has also been shown that, in the right circumstances, people can maintain performance relatively well, despite high levels of subjective fatigue (Ackerman, 2011). Notwithstanding the relevance of fatigue for real-life performance and well-being, many questions regarding its nature remain open. For example, there are many boundary conditions that play a role in whether – and if so how – fatigue will affect performance, such as the motivation to perform a task (Hopstaken et al., 2015a, b) and the spatial/temporal demands on processing task-relevant information (Borragán et al., 2017). Among the various cognitive operations involved in sustaining task performance, those that require top-down cognitive control seem to be especially sensitive to the detrimental effects of fatigue (Kok, 2022; Lorist, 2008; van der Linden et al., 2003). In line with this, we present two experiments that examine how the resolution of response conflicts during visually guided movement is altered under prolonged, fatiguing task conditions. Since the resolution of response conflict – enabling the selection of task-relevant responses while suppressing competing or automatically activated alternatives – is a central function of cognitive control, we assume that such task conditions place demands on cognitive control.

It is generally assumed that cognitive control is linked to a broad neural network in the prefrontal cortex that is able to orchestrate lower-level, often automatic perceptual, attentional, and motor processes, taking into account the task context and future goals (Miller, 2000; Stout, 2010). The role of cognitive control is therefore to ensure goal-oriented behaviour based on the synthesis of diverse sources of information available about the individual’s emotional-cognitive state, and the costs and benefits of the task involved. Compromised cognitive control due to fatigue requires compensatory effort to maintain adequate performance, and such effort may be supported by top-down attentional mechanisms that enhance the neural processing of task-relevant information, thereby improving discrimination between meaningful signals and distracting noise. In addition, compensatory effort can involve the active downregulation of neural activity, reflecting suppression of the fatigue-related system that typically produces negative feedback (Nakagawa et al., 2013). Compensatory efforts are particularly important when a task is considered valuable for achieving future goals or when immediate safety is at stake (Hockey, 2011). There is evidence that an adequate level of vigilance is also essential for effective cognitive control (Oken et al., 2006a, b). The most common definition of vigilance is the one that refers to the ability to sustain attention on a prolonged task performance (Parasuraman, 2000). Vigilance is known to play a key role in the effects of fatigue (Hancock, 2017). Accordingly, in the present study, we interpret vigilance as the ability to maintain attention, during trials involving a visually guided movement task over time-on-task.

It is well-known that vigilance is modulated by various neurotransmitter systems. The role of the locus coeruleus–norepinephrine (LC-NE) system seems to be especially critical as it determines both phasic and tonic vigilance and also seems to contribute to the compensatory responses under fatigue (Hopstaken et al., 2015a, b; Oken et al., 2006a, b). Longer response latencies of LC-NE neurons have been observed during poor performance and under reduced vigilance (Aston-Jones et al., 1994a, b; Oken et al., 2006a, b). Importantly, LC-NE activity seems to play a role both in sustaining tonic (long-term) vigilance levels and in phasic (short-term) vigilance adjustments in a stimulus-dependent manner (Rajkowski et al., 1994). For example, previous findings based on visual oddball tasks support the notion that LC-NE responses may underlie short-term fluctuations in vigilance through phasic responses, thereby enhancing the ability to orient toward unexpected stimuli (e.g. Rajkowski et al., 1994). This role is particularly relevant in our second experiment, in which the target location changes unexpectedly. In addition, longer-term changes in vigilance may be governed by variations in tonic LC-NE activity, which contribute to the maintenance of task-related attention. In fact, reduced activation of the LC-NE system underlies many facets of mental fatigue such as decreased vigilance, deteriorated cognitive control, and increased task disengagement (Aston-Jones et al., 1994a, b; Berridge et al., 1993; Hopstaken et al., 2015a, b, 2016, Oken et al., 2006a, b).

Prolonged task performance (or time-on-task) also seems to affect the preparatory and execution phases of motor responses. Both movement phases are demanding, and thus cognitive control processes are important for the precise, coordinated performance of movements (Janczyk & Kunde, 2010; Liu et al., 2008). In terms of the fatigue sensitivity of motor processes, Rozand et al. (2015) found that ToT increased the movement duration in arm pointing, when the pointing task was performed after another cognitively demanding task. In contrast, Solianik et al. (2018) observed the opposite: reaching movement towards a visual target accelerated and showed decreased variability with ToT. More recently, Matuz et al. (2022a, b) confirmed the fatigue-sensitivity of movement planning and execution. In three experiments, they found that simple pointing movements changed with ToT. In each experiment, they used a vigilance paradigm, which meant that the targets of the pointing movements appeared at widely different inter-trial intervals. These authors (2022) showed that both the planning and execution of motor responses were vulnerable to ToT. Based on examining different cueing conditions, they concluded, however, that ToT-related changes were not caused by phasic vigilance deficits, but rather by a reduced level of tonic vigilance.

The results of the studies outlined above leave open the question of exactly which aspects of cognitive control are sensitive to ToT and lead to reduced efficiency in movement performance. In this regard, one particularly important aspect is that cognitive control needs to ensure the stability of goal-directed behaviour in the presence of distractors (Mayr & Grätz, 2024). Ignoring distracting stimuli is especially demanding when distractors facilitate a different response to the appropriate one. In that case, response conflict occurs (van Veen & Carter, 2006; Verbruggen et al., 2006). It is known that the resolution of such response conflicts often deteriorates with fatigue (Csathó et al., 2012; Faber et al., 2012a, 2012b; Lorist et al., 2005). This conclusion, however, is derived from previous studies that have mainly focused on perceptual judgements or responses in simple reaction-time tasks. Nevertheless, these previous studies have not examined the fatigue sensitivity of response conflicts directly in relation to movement planning and execution. Since movement is an important component of goal-directed behaviour, studying the fatigue sensitivity of movement planning and execution brings us closer to understanding what processes may impede goal attainment in different environmental circumstances. Accordingly, to fill this knowledge gap, the two studies in the present paper explored the potential effects of fatigue (i.e. ToT) on response conflicts in tasks requiring simple pointing movements. Specifically, we investigated how fatigue, induced by ToT, modifies the resolution of response conflicts in visually guided pointing movements. Similarly to Matuz et al. (2022a, b), in two experiments, we employed a vigilance paradigm in both experiments: participants performed the pointing task continuously for a prolonged period without rest, while inter-stimulus intervals (foreperiods) varied widely, ranging from 500 to 7000 ms. This broad range of foreperiods imposes sustained attention and vigilance demands by inducing temporal uncertainty, which likely contributes to a faster ToT-related decline in performance (e.g. Matuz et al., 2024). We assumed that fatigue (particularly through the effect of reduced vigilance) would impair cognitive control resulting in deficits in movement performance under conflicting information about the direction of movements.

**Fig. 1:**
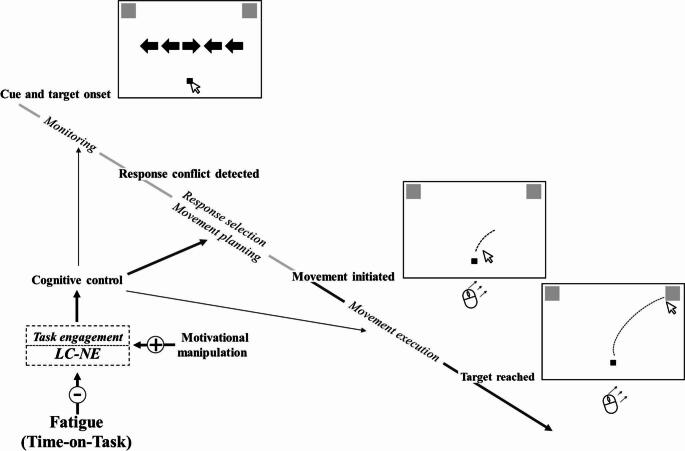
Concept of incongruent trials in Experiment 1. The figure illustrates the main cognitive steps associated with the preparatory and execution phases of visually guided pointing movements, as well as their sensitivity to fatigue, in the case of an incongruent trial. The preparatory phase lasts from the orienting cue indicating movement direction (central arrowhead) to movement initiation. Movement execution extends from movement initiation to target acquisition. The two phases are depicted as having approximately equal duration solely for visual clarity. Following the presentation of the cue and flanker stimuli, a monitoring process takes place. In incongruent trials, this is followed by the detection of response conflict and the planning of the movement trajectory. Movement initiation is inhibited until response selection and movement planning are completed. In the second phase, the pointing movement is executed toward the target. Time-on-Task induced Fatigue detrimentally affects cognitive control via a reduction in task engagement (indicated by a minus sign), which may be underpinned by reduced tonic and phasic activity of the LC-NE system. In this incongruent trial condition, cognitive control is most strongly involved during response selection (as indicated by the bold arrow). Increasing task-related motivation through a motivational message (see Methods of Experiment 1) may compensate for the negative effects of fatigue (indicated by a plus sign). Some conceptual elements and labels are adapted from Erb et al. (2021). LC-NE: locus coeruleus–norepinephrine system

In *Experiment 1*, the effects of response conflict on movements were tested using a mouse tracking version of the Eriksen flanker task adapted from Erb et al. (2021). The theoretical concept of Experiment 1 (i.e. for an incongruent trial) is schematized in Fig. 1. In each trial, an arrowhead centred on the screen and flanked by distractors (congruent, neutral, incongruent) indicated the direction of the target stimulus to which participants needed to point to with the cursor. Erb et al. (2021) emphasized the critical role of cognitive control in incongruent trials, proposing that the response conflict between the cue and the flankers is recognized by a monitoring process leading to a temporal pause of the motor output. This pause is intended to allow additional time for top-down resources to be mobilized in support of the controlled selection and movement planning process. This increases activation along a control-demanding pathway, thereby biasing response activations toward the task-appropriate motor response (Erb et al., 2021). Based on the presumed critical role of cognitive control, we expected fatigue-related changes during a prolonged performance of the flanker task. More specifically, we assumed that fatigue induced by ToT primarily manifests as a reduction in task motivation and the accompanying task disengagement. Task disengagement refers to a gradual withdrawal of attention and cognitive control from the ongoing task, typically occurring under prolonged task conditions. This effect is thought to predominantly impair cognitive processes that require greater effortful, top-down control (Yee & Braver, 2018). From this perspective, and with regard to the variables measured, we hypothesized that performance in incongruent trials – requiring greater cognitive control – would deteriorate with ToT, compared with congruent and neutral trials that place lower demands on cognitive control. We also expected that ToT-related deterioration in incongruent trials would primarily affect the planning phase (i.e., the initiation time of the pointing response), rather than the execution phase of the movement (see Fig. 1).

The present study differs from previous movement-related studies that have examined spatial attentional orientation under fatigue. Specifically, several previous studies have reported that spatial attentional orientation is associated with arousal and vigilance (or alertness): decreasing vigilance in time on a task has been accompanied by increasing rightward attentional bias (Fimm et al., 2006; Manly et al., 2005; Paladini et al., 2016). One plausible explanation of this ToT-induced bias is an altered balance between two functionally and anatomically distinct attention networks (Newman et al., 2013). While the bilateral orienting network seems to become active when focusing on stimuli across different spatial areas, the right-lateralized ventral attention network has a non-spatial function and mainly controls overall attentional capacity and vigilance. However, as fatigue induced by ToT increases, the right-lateralized attention network decreases in activation, and accordingly the left hemisphere lateralized part of the orienting network becomes more dominant (Newman et al., 2013). This enhanced dominance of hemisphere-controlled attention is manifested in a rightward attentional bias, which appears to be linked to a higher attentional shift cost from fixation toward peripherally presented targets (Paladini et al., 2016). In the context of the first experiment of this study, the rightward attentional bias makes it plausible to assume that the performance of leftward pointing movements will be impaired with increasing ToT compared to rightward movements. It is important to note, however, that the difference in the efficiency of rightward and leftward movements may not only be due to spatial attentional processes. Specifically, anatomical and biomechanical factors may lead to an advantage for leftward movements performed with the right hand (i.e. adductive movements) over rightward movements (i.e. abductive movements) (Bradshaw et al., 1988). This implies that in the present studies, pointing to the left target stimulus may be faster and more precise than movement toward the right stimulus. It may, therefore, be a question of the extent to which the biomechanical advantage of leftward movements compensates for the detrimental effects of attentional deficits induced by fatigue.

**Fig. 2:**
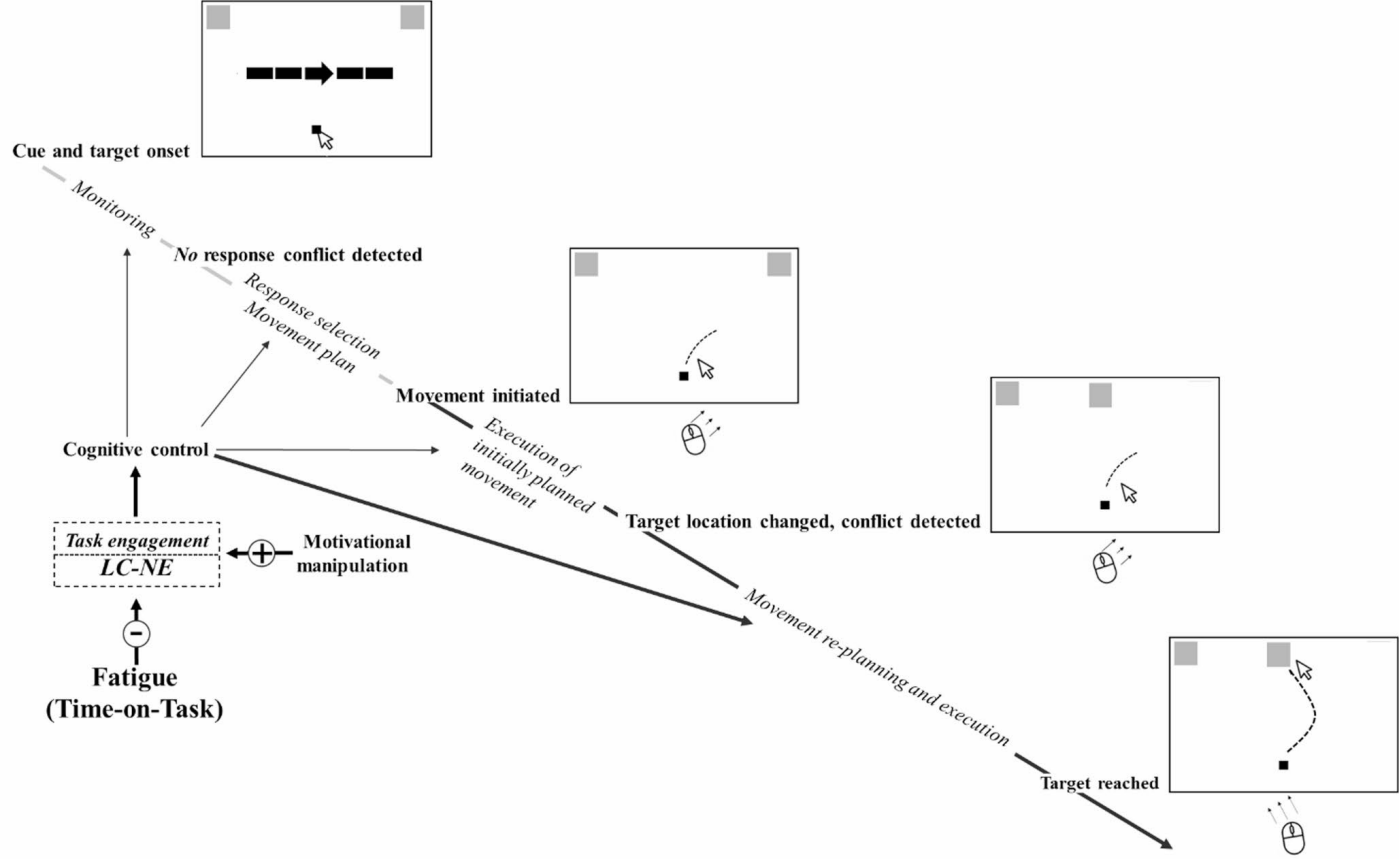
Concept of the leap trials in Experiment 2. During the preparatory phase of the movement, monitoring processes do not detect response conflict while inspecting a simple visual display. Therefore, response selection (i.e., choosing the correct movement direction) and movement trajectory planning require a lower level of cognitive control, as indicated by the thin arrows. Movement planning is followed by movement initiation and the execution of the initially planned movement, which also operates under lower level of cognitive control. In the trials of the second experiment, the target changes its position after movement initiation, creating a conflict between the initially planned movement direction and the direction required to reach the new target position. This response conflict is resolved through a cognitively demanding movement re-planning and execution process. However, increasing task-related motivation through motivational message (see the method of experiment 2) can compensate for the negative effects caused by fatigue (indicated by a plus sign). Some conceptual elements and labels of the figure are adapted from Erb et al. (2021). LC-NE: locus coeruleus-norepinephrine system

In *Experiment 2*, we examined the effects of response conflicts further with rare trials where the spatial location of the target stimulus unexpectedly changed after its appearance, with targets moving away from their initial location (a left or right peripheral location) to another one (a central location). In such trials, the unexpected change in target stimulus location created a conflict between the initially planned movement and the one required by the stimulus position (see also the schematic concept illustrated in Fig. 2). Resolving interference between an initiated and a required movement direction plausibly demands cognitive control processes such as response inhibition and flexibility. In the leap trials, cognitive control was needed to inhibit the originally planned movement and then replan the movement execution in accordance with the new target location. As suggested by previous studies, the control processes that enable such behavioural flexibility in particular may deteriorate as a result of fatigue (e.g. Matuz et al., 2019; van der Linden et al., 2003). Based on the above line of reasoning, we hypothesized that the execution phase of leap trials would worsen with increasing ToT.

Finally, in both experiments, we modified the level of task-related motivation after the ToT period. Specifically, through the presentation of a motivational message, we investigated whether the ToT-related performance deficits in pointing movements recovered as the task motivation increased. Several previous studies have shown that compromised performance due to fatigue can be largely attributed to task disengagement. The strong association between fatigue and disengagement can be explained by the role of fatigue as a key component of an integrated cost–benefit system that evaluates the rewards and energetic costs of performing a task, and thus fundamentally affects task-related motivation. As fatigue increases, it signals that the costs may outweigh the benefits, which facilitates the fatigued individual’s disengagement from the task (van der Linden, 2011). In other words, fatigue, task engagement, and cognitive control are dynamically related, with motivational processes potentially providing a feedback mechanism that can partially compensate for fatigue-related performance decline. Following this notion, it is evident that increases in motivation can reduce or even eliminate the performance deterioration in visually guided movements caused by ToT-induced fatigue (e.g. Hopstaken et al., 2015a, b, 2016).

The present study is novel in examining how mental fatigue induced by prolonged task performance selectively affects cognitive control processes during the planning and execution of visually guided movements, rather than simple perceptual decisions. By employing movement-based paradigms, the study bridges a gap between research on fatigue-related response conflict in reaction-time tasks and goal-directed motor behaviour. Also, the inclusion of motivational manipulation provides novel insight into how task engagement can partially compensate for fatigue-related performance declines in motor control tasks.

## Experiment 1

### Materials and methods

#### Participants

Thirty-two healthy volunteers participated in the first experiment. They were Hungarian undergraduate and postgraduate students recruited from the Faculty of Sciences and the Faculty of Humanities and Social Sciences at the University of Pécs, Hungary. Data on six participants were excluded due to an insufficient proportion of trials with valid fixations (< 80%) or because of stability problems with eye tracking, yielding a final dataset of 26 participants (15 females, aged between 20 and 30, *M* = 23.27, *SD* = 2.71).

The sample size was informed by prior research using similar ToT paradigms and task characteristics (Matuz et al., 2022a, b). In that study, three experiments tested 26, 25, and 24 participants, respectively, using a visually guided pointing task of comparable duration and structure. ToT effects reached moderate to large effect sizes on several movement variables (e.g., initiation time and movement time; η_p_² = 0.11 − 0.34). While these earlier findings provided a reference point for determining a comparable sample size, the present analyses relied on linear mixed models, which leverage all trial-level observations rather than only participant-level averages. This approach increases the effective sample size for estimating fixed effects and therefore provides reasonable statistical power to detect the effects of interest despite the limited sample size.

Assessed by the Edinburgh Handedness Inventory (15 items, brainmapping.org/shared/Edinburgh.php), participants were right-hand dominant or had an ambidextrous handedness, and each reported using the computer mouse with the right hand. All reported normal or corrected-to-normal vision and normal hearing, and none were taking medication. All participants provided written consent, and the study was approved by the United Psychological Research Ethics Committee (nr. 2023-58).

#### Task and stimuli

**Fig. 3:**
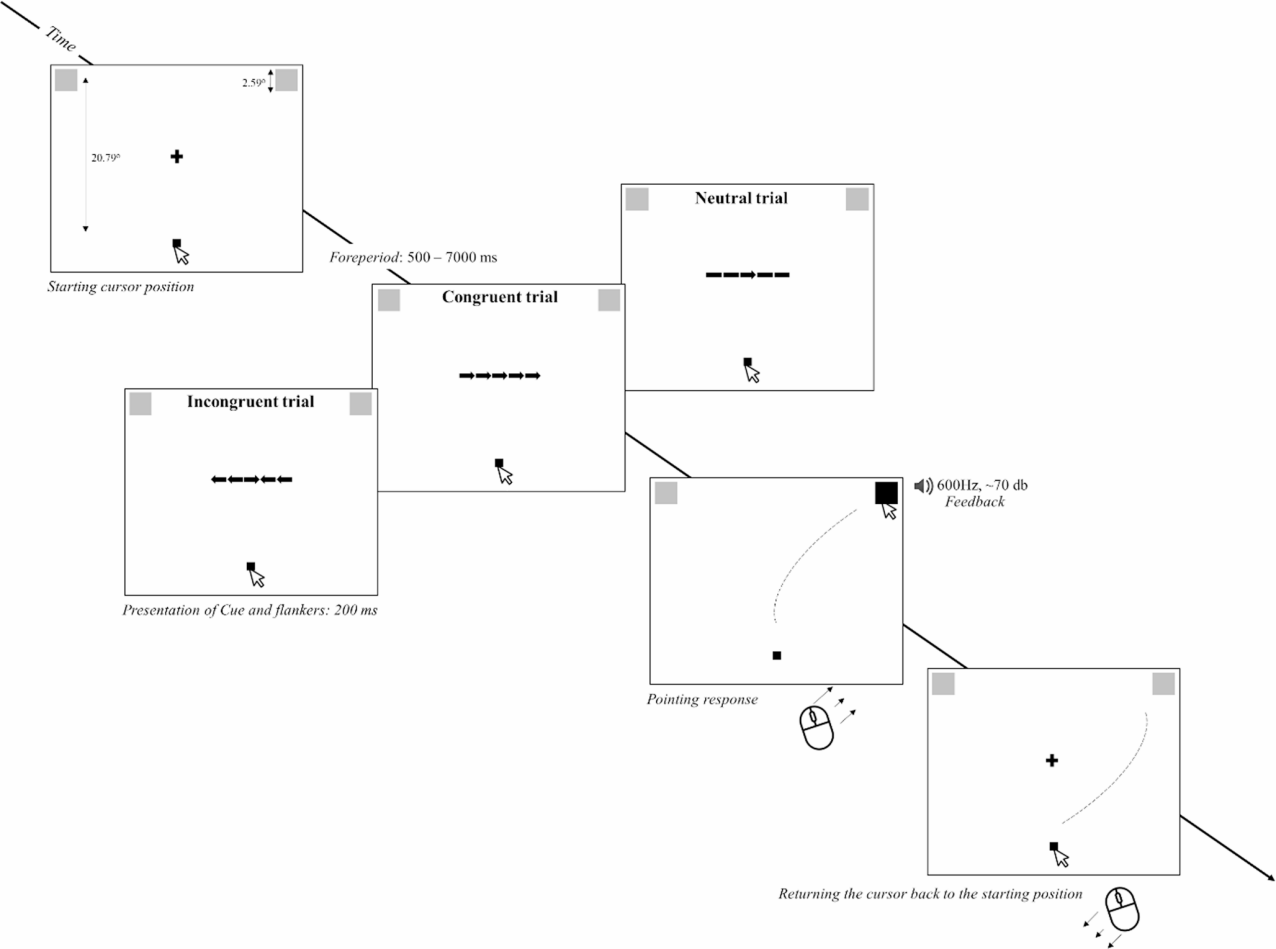
Schematized layout of the stimulus positions and the sequence of trials in Experiment 1. On each trial, participants performed a visually guided mouse-pointing task: they pointed to a target square by moving the cursor from the movement initiation point (the black square at the bottom of the screen) to the target. The target square (left or right square) was indicated by a centrally positioned black arrow (cue) flanked by distractors. The direction of the central cue was congruent, incongruent or neutral with the flankers

Figure 3 schematizes the sequence of a trial. Participants performed a visually guided mouse-pointing task programmed in PsychoPy 3 (version: 2023.2.2). Stimuli were displayed on a Tobii TX300 integrated monitor (23”), which had a resolution of 1920 × 1080 and a refresh rate of 60 Hz. The participants were seated ~ 60 cm from the screen with a standard computer mouse positioned for right-hand use. The background colour of the screen was white.

Two grey squares (100 × 100 pixels in size; 2.59^o^ x 2.59^o^; hex: #808080) were continuously presented in the left and right top corners of the screen. The centres of the left and right targets were located at [-800, 350] and [800, 350], respectively. Depending on the cue, either the left or right square served as the target stimulus. A black square (hex: #000000), 30 × 30 pixels (.81^o^ x .81^o^) in size, in the middle of the bottom of the screen (centre located at [0, -450]) was also continuously presented during the task. It was required to initiate mouse movements from this point and the start of a new trial was blocked if the mouse cursor was not located within the area of this square. The distance between the centre of the movement initiation point and the centre of the targets was 1131.37 pixels. Apart from the periods in which the cue and the flankers were presented, a fixation cross was present in the centre of the screen. Each trial started with a foreperiod ranging from 500 to 7000 ms, in steps of 500 ms. In each trial, the foreperiod duration was drawn from a uniform distribution, and for each participant, foreperiods were sampled from this fixed set and varied across trials. The same foreperiod sequence was preserved across blocks within the same individual to ensure within-subject comparability. The foreperiod ended with the presentation of the cue and flankers, which lasted 200 ms.

The cue stimulus was a centrally presented black arrow (65 pixels in length; 1.75^o^; hex: #000000) pointing either to the left or the right. In congruent trials, the flankers were four additional arrows in the same direction located next to the cue (two on the left and two on the right side of the cue). The flankers were identical to the cue except for their location. In incongruent trials, the flankers pointed to the opposite direction to the cue (e.g. if the cue pointed to the left, the flankers pointed to the right). In neutral trials, the presentation of the cue was accompanied by simple horizontal lines without arrows (two on both sides of the cue) instead of flankers.

Participants were instructed to look at the fixation cross until the cue and flankers appeared. Then they had to move the mouse cursor onto the target location as quickly and precisely as possible. Time and two-dimensional mouse coordinates were recorded continuously at 60 Hz, corresponding to the monitor’s refresh rate. The trial was successful if the cursor reached the target and remained within the target area for 100ms. Upon meeting these criteria, the colour of the target changed to black, and either a 600 Hz or a 300 Hz tone was played for 200 ms indicating whether the response was correct or incorrect, respectively. After each trial, the participants were required to move the cursor back to the movement initiation point (i.e. the black square presented at the bottom of the screen).

#### Procedure

**Fig. 4:**
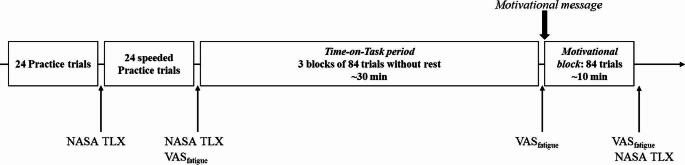
The schematized procedure of Experiment 1. Participants first performed two blocks of practicing trials. It was followed by the Time-on-Task period (ToT), when the participants performed 3 blocks of 84 trials without rest. When the ToT was ended then a motivation message appeared on the screen explaining that the experiment would end much earlier if they perform the task faster and more accurately than before. The message was followed by a block of 84 trials (Motivational block). Participants indicated their subjective fatigue (VAS_fatigue_) and workload (NASA-TLX) three times during the experiment

The procedure is schematized in Fig. 4. To avoid any mismatch between participants’ general daytime activity and the time when the experiment was conducted, prior to the experiment, participants’ preferred time for activity was assessed using the self-assessment version of the Morningness-Eveningness Questionnaire (MEQ-SA; Terman et al., 2005), and accordingly, participants were tested at the time of day that aligned best with their chronotype. Six participants were ‘evening types’ (MEQ-SA score ≤ 41), one was a ‘morning type’ (MEQ-SA score > 58) and 19 were ‘intermediate-types’ (MEQ-SA scores between 42 and 58). All of the participants were asked to get adequate sleep during the night prior to the experiment. The average sleep duration assessed by self-report was 7.46 h (*SD* = 0.99). Participants were instructed to abstain from alcohol for 24 h before the experiment and from caffeine 3 h beforehand. Participants were seated in a dimly lit, sound-attenuated room. After answering sleep-related and general questions, the task and the procedure were fully explained except for the motivation manipulation, and to ensure consistency, task instructions were presented on video. After the instructions, the eye-tracking device (Tobii TX300; sampling rate: 60Hz) was calibrated using a standard 9-point eye-tracking calibration procedure. A chin rest was used to enhance the accuracy of eye movement recordings and to standardize the eye position across participants. Trials where participants did not fixate on the fixation cross during the presentation of the stimuli were excluded from further analyses. The eye-tracking procedure was used for fixation control only, and so no further analysis of the eye-movement data was performed.

Participants practised the task in two parts. First, they performed 24 practice trials with parameters identical to those of the real task, while the second part of the practice included another 24 trials, but this time a warning message (i.e. “Faster!”) was presented if the participant did not respond within 1 s. These speeded trials were included to ensure that the participants followed the instructions regarding speed. After each practice block, participants completed the NASA Task Load Index (NASA-TLX; Hart & Staveland, 1988), which assessed the perceived level of workload in the following six domains: mental demand, physical demand, time demand, performance, effort and frustration. Each subscale was rated on a 21-point scale. After the practice session, the ToT period followed. During this period, participants performed three blocks of 84 trials without a break, lasting approximately 10 min each (~ 30 min in total). At the beginning of the ToT, participants were asked to indicate their subjective fatigue on a 100 mm-long visual analogue scale presented on the computer screen (“no fatigue at all” was presented on the left and “very severe fatigue” on the right side of the scale; pre-ToT rating). Within each block of the ToT, cue conditions and the side of target stimuli were balanced equally. Trials were presented in a pseudo-random order. When the ToT period ended, the participants again indicated their level of subjective fatigue on the visual analogue scale (post-ToT rating).

After the post-ToT rating, the *motivation manipulation* was introduced; that is, a message appeared on the computer screen for 20 s explaining that the time left in the experiment would depend on their task performance (i.e. speed and accuracy). Participants were informed that the experiment would end much earlier if they performed the task faster and more accurately than before. In reality, the duration of the following block of trials (henceforth, ‘motivational block’) was identical to the preceding blocks (i.e., approx. 10 min). All task parameters in this motivational block were identical to those of the preceding blocks. At the end of the motivational block, participants were asked again to indicate their subjective fatigue and to fill in the NASA-TLX as well.

#### Data analysis

##### Performance measures

Time-stamped mouse coordinates were first analysed using a Python script. The overall accuracy was very high (mean: 99.6%; range: 97.92% − 100%), and therefore it was not further analysed. Four performance measures were calculated and used for statistical analyses. Histograms were generated for each outcome variable, and cut-off thresholds were determined based on the observed distributions in order to approximate a normal distribution. To assess the movement preparatory phase, the *initiation time* (IT) was analysed. IT was calculated as the interval between the onset of cue presentation and movement initiation (i.e. moving the cursor ~ 15 pixels from the centre of the movement initiation point). Trials with ITs faster than 300 ms and slower than 800 ms were excluded from the analyses (0.22%). *Movement execution* was assessed via a temporal and a spatial metric. As a temporal metric, *movement time (MT)* was calculated to assess the temporal dynamics of a response. MT was defined as the interval between movement initiation and the time of reaching the target stimulus. Trials with MTs slower than 1,500 ms were excluded from the analyses (0.22%). As a spatial metric, the highest spatial deviation (HD) from the task axis (i.e. the shortest path to the target) was calculated. Trials with HDs higher than 300 pixels were excluded (1.16%). Non-horizontal cursor movements on a 2D surface, generated primarily through movements of the finger joints, can exhibit substantial variability. Accordingly, cursor movements, which are determined by numerous kinematic and biomechanical factors, often deviate from a straight line (Lee & Bang, 2013). In the present study, when defining HD, we used a straight-line reference (i.e. the shortest path to the target) for the sake of simplicity and to ensure comparability across participants. However, this also represents a limitation of our study.

##### Statistical analyses

Statistical analyses were carried out in RStudio (version 2024.04.2.) using the ‘afex’ (Singman et al., 2015), ‘ggplot2’ (version 3.42), ‘lme4’ (version 1.1–34), ‘lmerTest’ (version 3.1-3) and ‘emmeans’ (version 1.8.8; Lenth, 2024) packages. Changes in subjective fatigue were analysed using linear mixed models (LMMs) with Administration Time (i.e. three administrations of the visual analogue scale) as fixed effects and subject ID as a random effect. Changes in perceived workload were also analysed through LMMs, with Administration Time (three administrations of the NASA-TLX scales) and Subscale (i.e. the six NASA-TLX scales) as fixed effects and subject ID as a random effect.

To examine the effects of ToT on task performance, separate LMMs were fitted for each performance metric (i.e., IT, MT, RT, and HD). Importantly, cognitive control and task engagement may exhibit substantial inter-individual variability (Braver, 2012; Locke & Braver, 2008; Shaw et al., 2010; Hancock, 2017; Matthews et al., 2017). Therefore, when testing the possible linear mixed models, we initially specified a maximal random-effects structure in which all relevant explicit variables were included as random effects. In each model, the respective performance metric served as the outcome variable. Block (i.e. the three ToT blocks), Congruency (i.e. the three congruency conditions) and Side (i.e. left or right target) were entered as fixed effects (including interactions), while subject ID was entered as a random effect. To test the effects of the motivational manipulation, a very similar LMM was used except that the Block factor had two instead of three levels: the third block of ToT was compared with the motivational block. The model fitting process for LMMs was as follows. In each case, a full model including all sets of interactions as both fixed and random effects was first tested. The full model was specified as: Performance metric ~ Block * Congruency * Side + (Block * Congruency * Side|Subject ID). This specification estimates population-level (fixed) effects of Block, Congruency, and Side and all their interactions, while simultaneously allowing each participant to have their own baseline performance and their own subject-specific deviations for each effect, including interaction terms.

Following Barr et al. (2013), when the full model did not converge, it was systematically reduced by removing random effects until convergence was achieved. That is, we gradually reduced the complexity of the random-effects structure by first removing random slopes for interaction terms and, subsequently, random slopes for selected main effects, while keeping the fixed effects structure unchanged. More specifically, model reduction proceeded by first removing random slopes for interaction terms. Specifically, the three-way random slope (Block * Congruency * Side) was reduced to models containing random slopes for Block * Side or Block * Congruency. If these models still resulted in singular fits, the random-effects structure was simplified further by retaining only random slopes for the main effects (Block + Congruency + Side).

When necessary, additional reductions were performed by removing selected random slopes while retaining the random intercept for Subject. In these cases, models containing random slopes for Block + Side or Block + Congruency were tested. If singular fits persisted, models including only a single random slope (Block, Congruency, or Side) were fitted. Finally, if all previous models resulted in singular fits, a model with only a random intercept for Subject was retained. This reduction strategy prioritized retaining random slopes associated with Block and its interactions, as ToT effects represented the primary focus of the study, followed by Side and Congruency.

Explained variance was assessed by calculating marginal R^2^ and conditional R^2^, which indicate the variance explained by fixed effects and the whole model, respectively. Significant fixed effects and their interactions were followed up by contrast analyses using the ‘emmeans’ package. To obtain *p* values, Satterthwaite’s method was used to calculate degrees of freedom. Bonferroni correction was employed to adjust for multiple comparisons. For pairwise post-hoc comparisons, effect sizes were estimated using Cohen’s d.

Supplementary Figures S1 and S2, and S5–S8 depict participants’ subjective scores and cognitive performance over time. Estimates derived from linear mixed models are reported in Supplementary Tables S1 and S2. Supplementary Table S5 lists the final formulas of all linear mixed models that successfully converged. Conditional residual plots from the LMM analyses for each subjective (i.e. subjective fatigue and perceived workload; Figure S17) and performance measure (Figure S18) are also presented in the Supplementary material. In addition, we report the correlations between the random effects estimated by the LMM analyses (Supplementary material, Table S6).

### Results

#### Subjective fatigue and workload

**Fig. 5:**
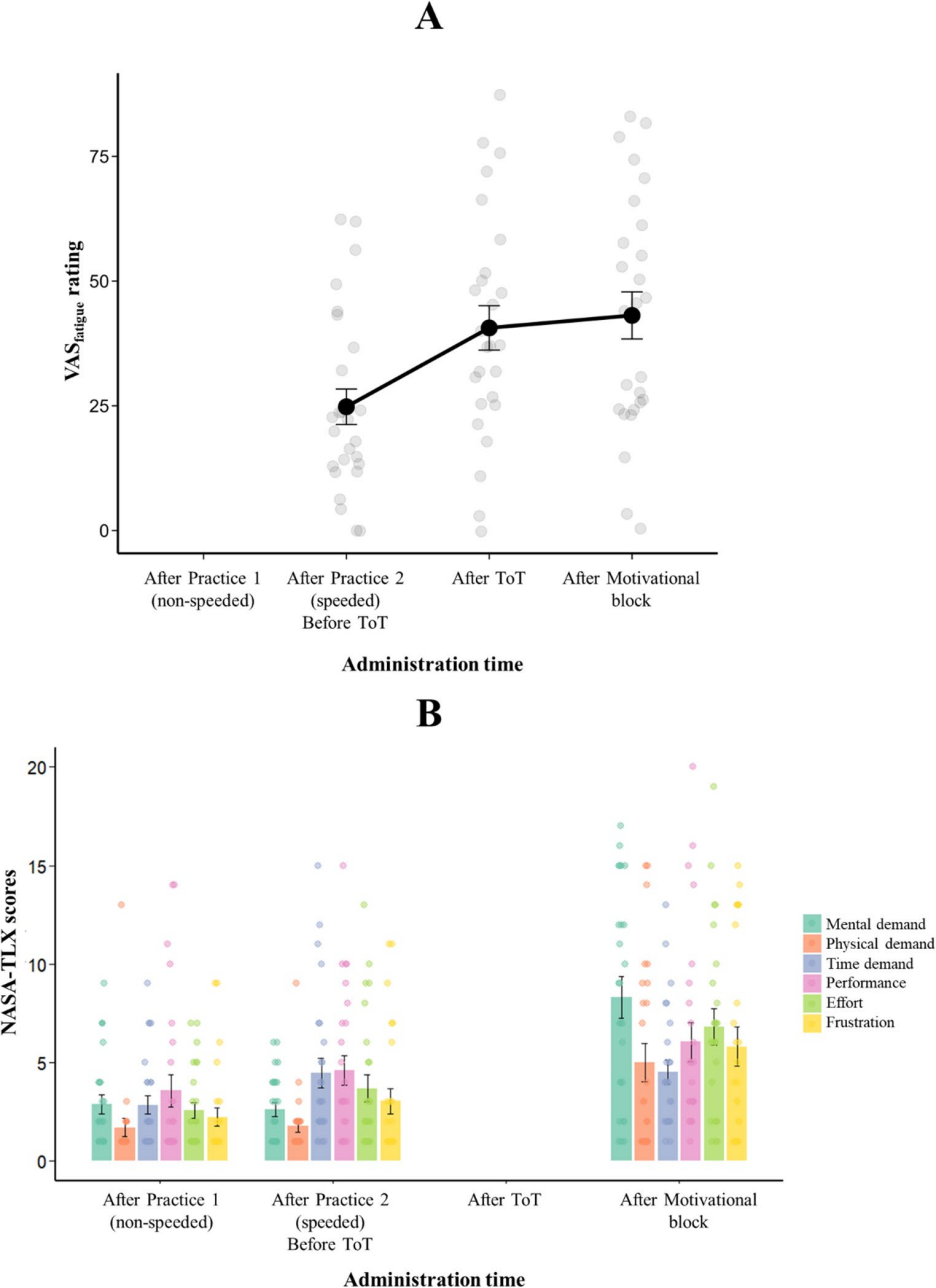
Results of the analysis of subjective fatigue (**A**) and perceived workload (**B**) in Experiment 1. Black dots (A) and coloured bars (B) represent average ratings/scores for each Administration time; error bars represent the standard error. Gray (A) and coloured dots (B) indicate individual ratings. ToT: Time-on-Task. The blank bar positions indicate that the measure (VAS_fatigue_ or NASA-TLX) was not administered at that time point

The results of subjective fatigue and workload are presented in Fig. 5. The analysis of subjective fatigue (marginal R^2^ = 0.12, conditional R^2^ = 0.62) yielded a significant Administration time (i.e. three administrations of the visual analogue scales) main effect (F(2, 50) = 12.53, *p* < .001). Bonferroni-corrected post-hoc analyses showed that subjective fatigue significantly increased after the ToT (pre- vs. post-ToT ratings: t(50) = -3.99, *p* < .001, Cohen’s *d* = -1.11, pre-ToT rating: mean = 24.81, SD = 18.14; post-ToT rating: mean = 40.62, SD = 22.70) but did not change after the motivational block (post-ToT rating vs. post-motivational block rating: t(50) = -0.63, *p* = 1.00, Cohen’s *d* = − 0.18; post-motivational block rating: mean = 43.12, SD = 24.05). The analysis of NASA-TLX scores (marginal R^2^ = 0.20, conditional R^2^ = 0.53) also yielded a significant Administration time (i.e. three administrations of NASA-TLX) main effect (F(2,425) = 67.21, *p* < .001). Post-hoc analyses indicated that perceived workload increased significantly after the experiment compared to the first (t(425) = -11.00, *p* < .001, Cohen’s *d* = -1.25) and second (t(425) = -8.67, *p* < .001, Cohen’s *d* = -0.98) practice blocks. The two practice blocks did not differ significantly (t(425) = -2.34, *p* = .06, Cohen’s *d* = -0.26). There was also a significant Subscale main effect (F(5,425) = 5.17, *p* < .001). Physical demand was rated significantly lower than mental demand (t(425) = -4.03, *p* < .01, Cohen’s *d* = -0.65; performance (t(425) = -4.35, *p* < .001, Cohen’s *d* = − 0.70; and effort (t(425) = -3.48, *p* < .01, Cohen’s *d* = − 0.56). Finally, the Administration time x Subscale interaction was also significant (F(10,425) = 3.33, *p* < .001). Post-hoc analyses showed that ratings on each subscale increased from the two practice blocks to the post-experiment administration (all t-values > 3.61, *p-*values < 0.01, all Cohen’s *d-*values > 1.00) except for time demand, which showed no significant change (all t-values < 2.20, *p*-values > 0.56, all Cohen’s *d* < 0.59), and performance, which only increased from the first practice block to the post-experiment administration (t(425) = 3.21, *p* = .026, Cohen’s *d* = − 0.89).

#### Changes in performance measures with increasing time-on-task

**Fig. 6:**
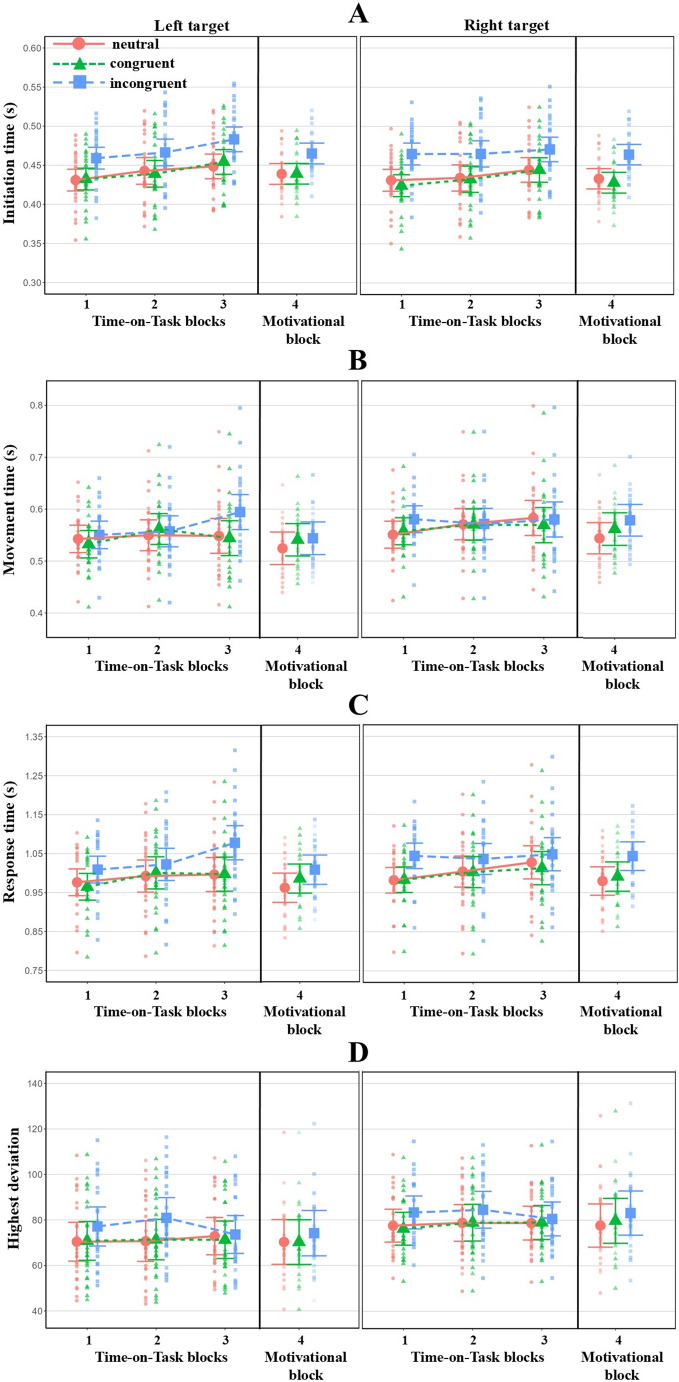
Model-predicted marginal means from LMM analyses for four performance measures (**A**–**D**) in Experiment 1. Error bars represent the 95% confidence intervals. The individual data points represent the predicted within-subject average for each block / trial condition

Performance measure results are presented in Table 1; Fig. 6. Here we only report the most important findings. The Congruency main effect was significant for all four performance measures. Post-hoc analyses revealed that, compared to both neutral and congruent trials, performance was significantly lower in incongruent trials (all t-values > 2.95, all *p*-values < 0.01, all Cohen’s *d-*values > 0.08); that is, participants initiated and executed their responses more slowly and with a greater deviation from the ideal path. The neutral and congruent trials did not differ significantly in any of the four measures (all t-values < 0.63; all *p*-values = 1.00, all Cohen’s *d-*values < 0.03). The Side main effect was significant for IT, MT, and HD, but only marginally significant for RT. In contrast to the rightward movements, the leftward movements were characterized by a slower preparatory phase (slower IT), followed by a faster (MT) and more precise (HD) execution. RTs tended to be slower when a response to the right target was required. Thus, participants took longer to prepare leftward responses, but once they had executed the movement, they were faster and more accurate.

**Table 1:** Main effects and interactions by LMM for ToT-related changes in Experiment 1

	Block (B)	Cong. (C)	Side (S)	B x C	B x S	C x S	B x C x S
Initiation time							
df	25.00	5969.38	24.97	5969.06	5971.13	5969.11	5969.09
F	11.23	294.09	8.56	1.28	4.12	1.91	2.32
*p*	< 0.001	< 0.001	0.007	0.274	0.016	0.148	0.054
Marginal **R**^**2**^	0.069						
Conditional **R**^**2**^	0.459						
*Movement time*							
df	24.77	5975.39	24.79	5975.25	5978.00	5975.28	5975.46
F	1.96	6.57	11.05	1.83	0.26	0.74	2.44
*p*	0.163	0.001	0.003	0.121	0.771	0.479	0.045
Marginal **R**^**2**^	0.009						
Conditional **R**^**2**^	0.148						
*Response time*							
df	25.18	5972.13	24.87	5971.93	5974.29	5971.97	5972.10
F	4.36	48.79	3.79	1.64	0.96	0.42	3.63
*p*	0.024	< 0.001	0.063	0.162	0.382	0.655	0.006
Marginal **R**^**2**^	0.021						
Conditional **R**^**2**^	0.246						
*Highest deviation*							
df	24.71	5922.51	24.75	5922.10	5922.60	5921.23	5922.33
F	0.31	8.96	7.42	1.15	0.02	0.15	0.23
*p*	0.737	< 0.001	0.012	0.330	0.982	0.864	0.924
Marginal **R**^**2**^	0.008						
Conditional **R**^**2**^	0.125						

LMM: linear mixed model; df: denominator df

The Block (i.e. the three ToT blocks) main effect was significant for IT and RT only. Post hoc analyses revealed that IT was significantly higher in the third block than in the first (t(25) = 4.08, *p* < .01, Cohen’s *d =* 0.38) and second (t(24.9) = 3.59, *p* < .01, Cohen’s *d =* 0.24) blocks, and that RT in the third block was significantly higher than in the first block (t(25) = 2.92, *p* < .01, Cohen’s *d =* 0.2). The analysis of IT also showed a significant Block x Side interaction. Follow-up analyses indicated two potential sources of this interaction. First, in trials requiring movements to the left, ITs were significantly higher in the third block than both in the first (t(31.1) = 4.78, *p* < .001, Cohen’s *d =* 0.47) and second block (t(37.2) = 3.74, *p* < .01, Cohen’s *d =* 0.28), while in trials requiring rightward movements, only the first block differed significantly from the third one (t(31.1) = 2.95, *p* = .04, Cohen’s *d = − .2*9). Second, the post hoc contrasts also revealed that leftward movement initiation was significantly slower than rightward, but only in the third block (t(89.1) = 3.71, *p* < .01, Cohen’s *d =* 0.20). There was also a significant Block x Congruency x Side interaction for RT and MT, but it was only marginally significant for IT. Follow-up analyses of RT revealed that it increased significantly over time, but only in incongruent trials requiring leftward movements; that is, RTs in left incongruent trials were significantly higher in the third block than in the first (t(106) = 4.18, *p* < .01, Cohen’s *d =* 0.41) and second (t(255) = 3.87, *p* < .01, Cohen’s *d = −* 0.33) blocks. All other contrasts were non-significant (Bonferroni-corrected *p*-values ranged between 0.11 and 1.00). Follow-up analyses of MT showed a very similar, although marginally significant trend. Specifically, the post-hoc comparisons revealed that MT in left incongruent trials was higher in the third block than in the the first (t(161) = 3.03, *p* = .051, Cohen’s *d =* 0.28) and second (t(414) = 2.84, *p* = .08, Cohen’s *d =* 0.23) blocks; however, this descriptive effect falls short of significance. Further follow-up analyses showed that MTs in incongruent trials differed significantly from congruent (t(5973) = 4.08, *p* < .001, Cohen’s *d =* 0.31) and neutral (t(5977) = 3.74, *p* < .01, Cohen’s *d =* 0.29) trials in the third block when leftward movements were required. All other contrasts were non-significant (Bonferroni-corrected *p*-values ranged between 0.27 and 1.00).

Follow-up analyses of the marginally significant three-way interaction for IT also exhibited a similar pattern regarding left incongruent trials (block 1 vs. 3: t(63.3) = 4.56, *p* < .001, Cohen’s *d =* 0.53; block 2 vs. 3: t(113.3) = 3.77, *p* < .01, Cohen’s *d =* 0.37). IT, however, also showed a significant increase over time on left congruent trials (block 1 vs. 3: t(61.5) = 4.16, *p* < .01, Cohen’s *d = −* 0.48; block 2 vs. 3: t(108.7) = 3.46, *p* = .01, Cohen’s *d = −* 0.34), left neutral trials (block 1 vs. 3: t(61.2) = 3.35, *p* = .03, Cohen’s *d = − .2*9) and right congruent trials (block 1 vs. 3: t(62.8) = 3.80, *p* < .01, Cohen’s *d = −* 0.44).

To further characterize the retained random-effects structure, we inspected the correlations among the random-effects estimates of the final models (see Supplementary Tables S6). Baseline performance (i.e., performance in the first block) showed only weak associations with ToT effects (median = 0.22, range = − 0.38 to 0.34). Thus, early task performance did not appear to systematically predict fatigue-related changes in performance. The random slopes of Block 2 (i.e., performance change from Block 1 to Block 2) and Block 3 (i.e., performance change from Block 1 to Block 3) showed strong positive correlations (median = 0.80, range = 0.61 to 0.91). These suggest that performance changes over time were consistent across individuals.

#### Motivation-related changes in performance measures

Table 2 summarizes the results of LMMs that test the motivational block-related changes in performance. Below we summarize the most important findings. After the motivational manipulation, cognitive performance improved significantly in terms of IT, MT, and RT; that is, after the manipulation, participants initiated and executed their movements faster than in the third block of the ToT period. The Congruency effect was significant for IT and RT, while it was marginally significant for MT. Similarly to the ToT period, ITs and RTs were significantly higher in incongruent trials than in congruent (IT: t(1968) = 10.34, *p* < .001, Cohen’s *d =* 0.57; RT: t(1978) = 4.72, *p* < .001, Cohen’s *d =* 0.26) and neutral (IT: t(1964) = 10.82, *p* < .001; RT: t(1971) = 5.27, *p* < .001, Cohen’s *d =* 0.28) trials. Finally, the Side effect reached significance for all four measures: participants initiated their movements faster toward the right target. However, MT and RT were slower, and HD was higher when movements had to be executed to the right side. None of the two-way and three-way interactions reached the level of significance. Correlations among the random-effects estimates of the final models indicated stronger motivation-related effects for participants with poorer performance in the last block of the ToT phase (median = − 0.82, range = − 0.86 to − 0.64; see Supplementary Table S6).

**Table 2:** Main effects and interactions by LMM for motivation-related changes in Experiment 1

	Block (B)	Cong. (C)	Side (S)	B x C	B x S	C x S	B x C x S
Initiation time							
df	25.23	1966.15	1966.47	1965.87	1964.18	1966.17	1968.14
F	4.38	74.22	11.70	0.97	0.47	1.95	0.32
*p*	0.046	< 0.001	< 0.001	0.380	0.495	0.143	0.727
Marginal **R**^**2**^	0.060						
Conditional **R**^**2**^	0.352						
*Movement time*							
df	25.12	1978.31	1977.79	1978.17	1973.97	1979.52	1981.8
F	6.22	2.81	11.58	1.45	0.04	0.21	1.26
*p*	0.020	0.061	< 0.001	0.235	0.833	0.809	0.285
Marginal **R**^**2**^	0.014						
Conditional **R**^**2**^	0.171						
*Response time*							
df	25.21	1975.05	1974.56	1974.73	1971.00	1975.82	1978.47
F	7.22	16.67	5.25	0.98	0.10	0.51	1.42
*p*	0.013	< 0.001	0.022	0.374	0.748	0.602	0.241
Marginal **R**^**2**^	0.023						
Conditional **R**^**2**^	0.245						
*Highest deviation*							
df	1960.58	1962.14	1961.89	1962.28	1962.72	1962.85	1962.26
F	0.16	0.87	16.80	0.28	0.00	0.33	0.07
*p*	0.692	0.417	< 0.001	0.755	0.984	0.719	0.930
Marginal **R**^**2**^	0.009						
Conditional **R**^**2**^	0.128						

LMM: linear mixed model; df: denominator df

### Discussion

The subjective fatigue and workload measures in Experiment 1 clearly showed that, over ToT, participants experienced more subjective fatigue, and their perceived mental load increased. The cue-flanker congruency affected all performance measures, thereby replicating previous studies that showed congruency effects in pointing movements (Erb et al., 2021). Specifically, compared to the congruent and neutral conditions, participants in the incongruent trials initiated the pointing movement more slowly, followed by a slower and more erroneous execution of movement. In other words, the congruency effect not only occurred in the preparatory phase of the movements but also compromised the execution of the movements. A similar effect was reported by Erb et al. (2018, 2021), with the difference that in their trials both the cue and the flankers remained visible during the movement execution. Therefore, the findings of Experiment 1 are particularly interesting, as cue and flanker stimuli were no longer visible during movement execution, yet the congruency effect persisted. This finding implies that the response conflict associated with incongruency, which made the preparatory phase difficult, was not resolved by the time the movement was initialized but persisted even after participants had initialized the movement towards the target.

Importantly, in line with a previous study (Matuz et al., 2022a, b), ToT had a significant and detrimental influence on movement initiation, and response time: overall, over time, fatigued participants prepared and performed their movements more slowly. However, the incongruency effect did not change with increasing ToT, thus our hypothesis was not confirmed.

With regard to differences between leftward and rightward movements, and in line with our assumption, we found that leftward movements exhibited a longer planning phase overall (i.e. slower initiation times), which was primarily driven by greater slowing of initiation times for leftward responses as a function of ToT. This finding may be explained by the compromised functioning of the right-lateralized attention network with increasing ToT. Such impairment could reduce the efficiency of attentional shifts from fixation toward targets presented on the left, thereby delaying the initiation of target-directed movements (Paladini et al., 2016). This finding is consistent with previous studies showing that fatigue can be accompanied by a rightward attentional bias (Fimm et al., 2006; Manly et al., 2005; Benwell et al., 2013; Paladini et al., 2016). In addition, rightward movements were less effective than leftward ones caused possibly by the biomechanical constraints of rightward arm movements (see also the Introduction).

The execution (i.e. MT) of incongruent leftward movement trials was also impaired as a function of ToT (although there was only a marginal effect on IT). In other words, a greater incongruency effect with increasing ToT was observed only when movement execution was directed toward the left target – a finding may reflect the combined influence of two factors. First, as noted above, the response selection process likely had not concluded by the time movement execution was initiated but continued during the execution phase and demanded cognitive control. Second, under fatigue (i.e. toward the end of the ToT period), leftward movements had an attentional disadvantage. Together, these two factors may explain why leftward movements in incongruent conditions exhibited performance deterioration with increasing ToT.

Finally, the results are in line with the motivational concept of mental fatigue, as, after the motivation manipulation, an overall improvement in performance speed was observed in three performance measures (i.e. IT, MT, and RT). The success of the motivational manipulation also indicates that during the Time-on-Task period, fatigued individuals showed disengagement, which in turn led to the slowing of pointing movement speed. Increased task motivation induced by the manipulation, however, did not compensate for the detrimental effect of incongruence on movements towards the left targets; that is, it did not affect changes in attentional bias related to fatigue.

In Experiment 1, cognitive control was needed to maintain goal-directed movement in the presence of distractors under increasing fatigue. In contrast, Experiment 2 elaborates on fatigue sensitivity by introducing another important aspect of cognitive control: in the rare leap trials (see below), a change in the initially planned direction of movement was required.

## Experiment 2

### Materials and methods

#### Participants

Thirty-one healthy new volunteers participated in the second experiment. As in Experiment 1, they were Hungarian undergraduate and postgraduate students recruited from the Faculty of Sciences and the Faculty of Humanities and Social Sciences at the University of Pécs, Hungary. Data on two participants were dropped due to an insufficient proportion of trials with valid fixations (< 80%) yielding a final dataset of 27 participants (22 females, aged between 20 and 26, M = 20.33, SD = 2.11). Assessed by the Edinburgh Handedness Inventory (15 items, www.brainmapping.org/shared/Edinburgh.php), one participant was left hand dominant and 26 had right-hand dominance or ambidextrous handedness. Each participant reported using the computer mouse with the right hand. All reported normal or corrected-to-normal vision and normal hearing, and none were taking medication. All participants provided written consent.

#### Task and stimuli

**Fig. 7:**
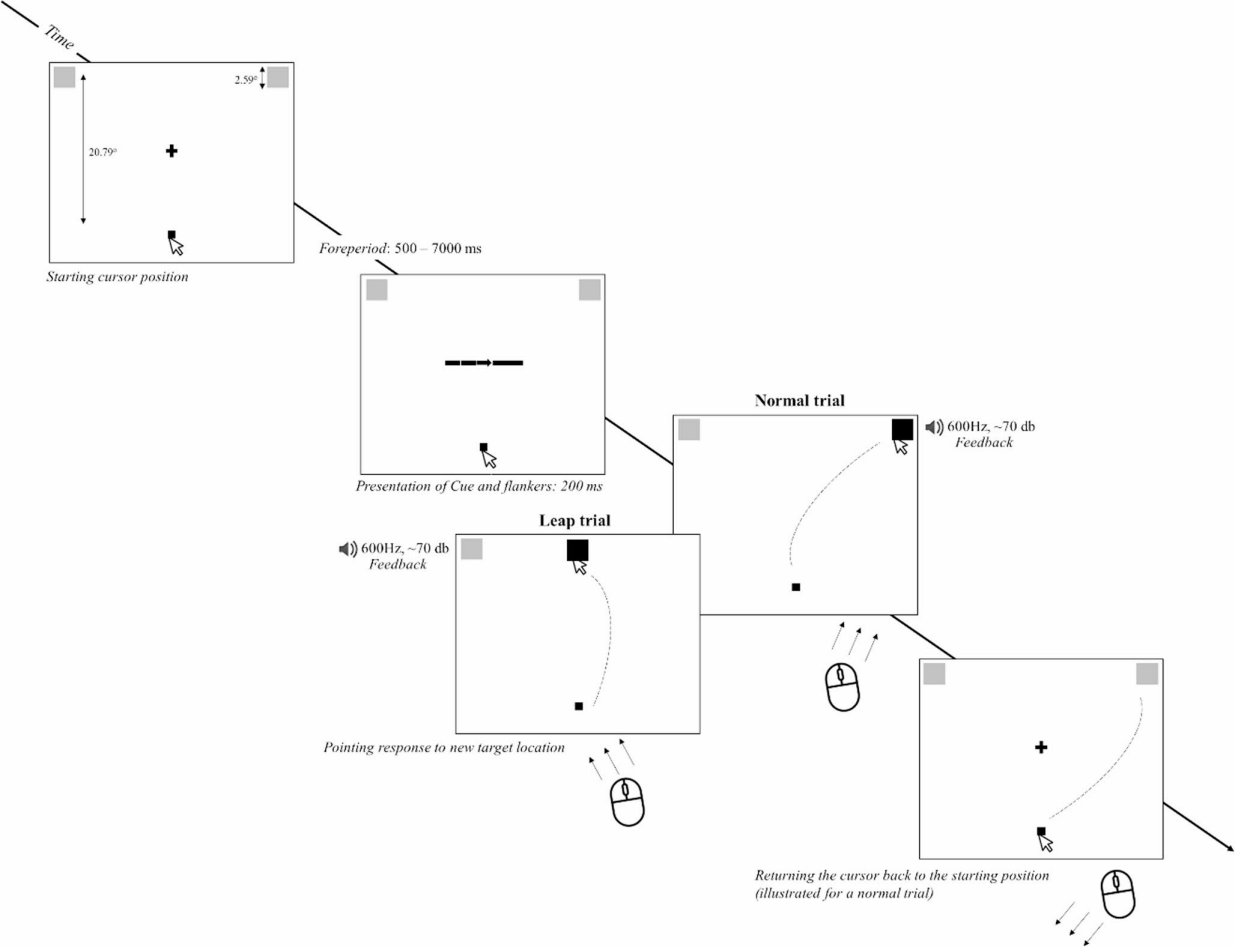
Schematized layout of the stimulus positions and the sequence of trials in Experiments 2. On each trial, participants performed a visually guided mouse-pointing task: they pointed to a target square by moving the cursor from the movement initiation point (the black square at the bottom of the screen) to the target. The target square (left or right square) was indicated by a centrally positioned black arrow (cue). In normal trials, there was no change in the target location during the trial: the target stimulus remained in the location where it was before the movement was initiated. In ‘leap trials’, the target stimulus changed its location right at the moment when participants initiated the cursor movement: the target stimulus was removed and, on the next frame, presented again in the top center of the screen. Participants needed to move the mouse cursor to the new target location

The experimental task was similar to that of Experiment 1. As in the first experiment, the target stimuli and the movement initiation point were continuously presented, and from the beginning of each trial, the mouse cursor had to be kept on the movement initiation point at the bottom of the screen (see Fig. 7). All parameters of the stimuli, the movement initiation point and the fixation cross as well as the duration of the foreperiod were identical to those of Experiment 1. In each trial, after the foreperiod, a black arrow stimulus flanked by horizontal lines without an arrowhead (identically to the neutral condition in Experiment 1) pointing either to the left or to the right was centrally presented for 200 ms. The task was identical to that of Experiment 1.

Two trial conditions were created: normal and leap trials. In normal trials, the target stimulus remained in the location where it was visible before the movement was initiated, i.e. the target location did not change during the trial. In contrast, in ‘leap trials’ (a new trial condition compared to Experiment 1), the target stimulus changed its location after movement initiation. More specifically, right at the moment when participants initiated the movement (i.e. the cursor left the movement initiation point) the target stimulus was removed and, in the next frame, was presented again in the top centre of the screen (centre of the target located now at [0, 350]). Participants then needed to move the mouse cursor to the new target location. All other task parameters remained the same as in normal trials. Approximately 9% of all trials were only leap trials. The aim of the infrequent presentation of the leap trials was to minimize the anticipation of the change in stimulus location and thus maximize the extent to which movement toward the new target location required flexible replanning of the initial movement plan. The occurrence of leap trials was equally balanced across blocks, with each block consisting of 80 normal and eight leap trials. Trials were presented in a pseudorandom order. After a leap trial, the three subsequent trials were always normal ones to minimize the effect of pre-planning of responses for the sudden change in the location of the targets.

#### Procedure

The procedure of Experiment 2 was identical to that of Experiment 1. Participants performed two blocks of practice trials that included not only normal trials but also two leap trials in each block. After the practice trials, the participants performed three blocks of 88 trials (ToT period; 264 in total, lasting ~ 30 min) without rest. Then the same motivational message as in Experiment 1 was presented followed by an additional block of 88 trials.

#### Data analysis

##### Performance measures

The same performance measures were calculated as in Experiment 1. For the calculation of HD in the case of leap trials, the task axis (i.e. the ideal path to the target) was determined as the straight line between the movement initiation point and the new location of the target. Exclusion criteria for the performance metrics were identical to those of the first experiment, except for HD (excluded trials: 1.15% (IT), 0.42% (MT), 0.69% (RT)). In Experiment 2, no limits were applied for HD due to the in-trial changes of the task axis in leap trials. As in Experiment 1, the overall accuracy was very high (mean: 99.26%; range: 96.30% – 100%) and was not further analysed.

##### Statistical analyses

The analyses of subjective fatigue ratings and NASA-TLX scores were identical to Experiment 1. Performance measures were analysed in two separate sets of analyses. First, to test the effects of the side of target stimulus presentation and whether it was moderated by ToT, LMM was used with within-subject factors Block (i.e. three blocks of trials) and Side (i.e. left vs. right) and subject ID as a random effect. Thus, the full model was specified as Performance metric ~ Block * Side + (Block * Side|Subject ID). Similar to Experiment 1, the systematic reduction of model complexity involved removing the interaction term first, then including only a single random slope (Block or Side). If all previous models resulted in singular fits, the model was reduced to a random-intercept-only structure for Subject ID. In these analyses, leap trials were excluded because, for the estimation of the Side effect in leap trials, the number of trials was too low (i.e. only 4 trials per block for left-, and right-sided targets). For the motivation-related changes, the Block factor included the last block of the ToT-period and the motivational block, while the other parameters remained the same (see Experiment 1).

Second, to test the effects of leap trials, and whether their effect is moderated by ToT, LMM was used with within-subject factors Block (i.e. three blocks of trials) and Condition (i.e. leap trial vs. normal trial) and subject ID as a random effect. The full model, therefore, was specified as Performance metric ~ Block * Trial type + (Block * Trial type|Subject ID). Similar to the first set of analyses, model complexity was reduced systematically by first removing the interaction term from the random effects structure, followed by models including only a single random slope (Block or Trial type). Again, if all previous models resulted in singular fits, the model was reduced to a random-intercept-only structure for Subject ID. To account for potential confounding effects resulting from the limited variance in the foreperiod preceding leap trials, in these analyses, only non-leap trials with a foreperiod of between 2.5 and 3.5 s were included. Thus, in these analyses, leap and normal trials did not differ in terms of the foreperiod. Supplementary Figures S3 and S4 and S9–S16 depict participants’ subjective scores and cognitive performance over time. Estimates derived from linear mixed models are reported in Supplementary Tables S3 and S4. Supplementary Table S5 lists the final formulas of all linear mixed models that successfully converged. Conditional residual plots from the LMM analyses for each subjective (i.e. subjective fatigue and perceived workload; Figure S19) and performance measure (Figures S20 and S21) are also presented in the Supplementary material. In addition, we report the correlations between the random effects estimated by the LMM analyses (Supplementary material, Tables S7 and S8).

### Results

#### Subjective fatigue and workload

**Fig. 8:**
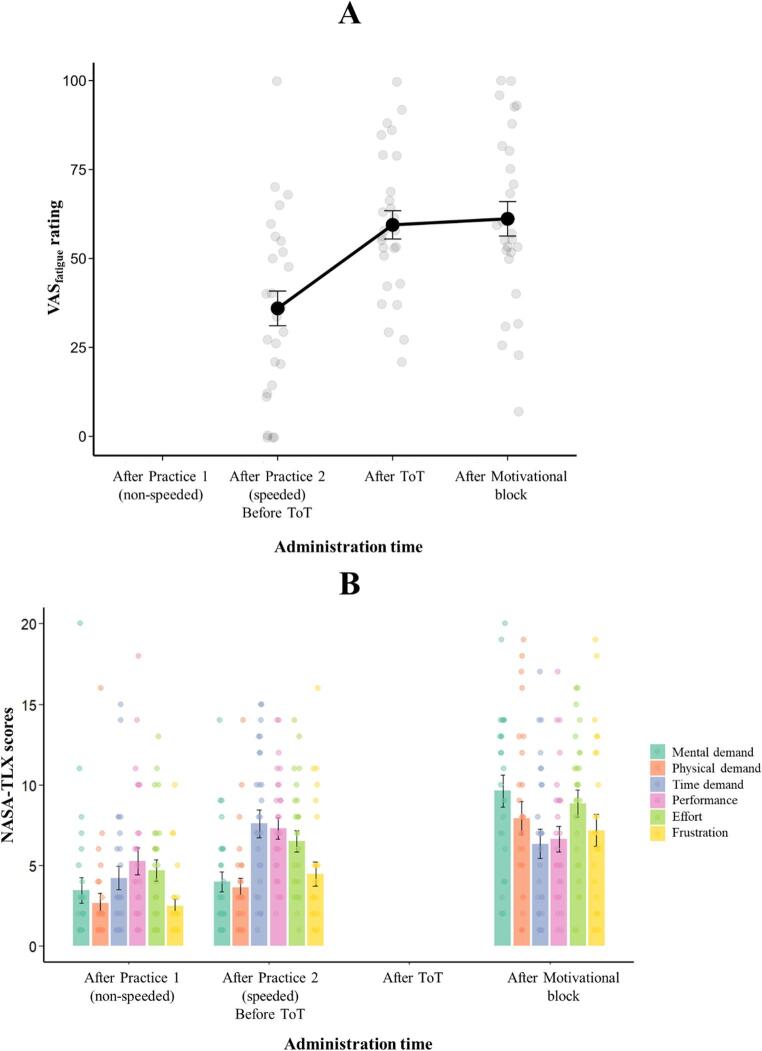
Results of the analysis of subjective fatigue (**A**) and perceived workload (**B**) in Experiment 2. Black dots (A) and coloured bars (B) represent average ratings/scores for each Administration time; error bars represent the standard error. Gray (A) and coloured dots (B) indicate individual data. ToT: Time-on-Task. The blank bar positions indicate that the measure (VAS_fatigue_ or NASA-TLX) was not administered at that time point

The results for subjective fatigue and workload are presented in Fig. 8. Similarly to Experiment 1, ToT led to a significant increase in subjective fatigue (Time main effect: F(2,52) = 15.92, *p* < .001, marginal *R*^*2*^ = 0.19, conditional *R*^*2*^ = 0.52; pre vs. post-ToT ratings: t(52) = -4.71, *p* < .001, Cohen’s *d* = -1.28), while the ratings after the motivational block did not differ significantly from the post-ToT ratings (t(52) = − 0.34, *p* = 1.00, Cohen’s *d* = − 0.09).

The Administration time (i.e. three administrations of the NASA-TLX scales) main effect was significant for NASA-TLX scores (F(2,26) = 22.30, *p* < .001, marginal *R*^*2*^ = 0.21, conditional *R*^*2*^ = 0.54). Post-hoc analyses showed that the perceived workload of the prolonged task performance was significantly higher than that of the first (t(26) = 6.40, *p* < .001, Cohen’s *d* = -1.28) and second (t(26) = 3.75, *p* < .01, Cohen’s *d* = − 0.71) practices suggesting that workload was enhanced by ToT. Importantly, the analyses also revealed that scores on the Mental demand subscale increased significantly from the two practice blocks to the post-experiment administration (all t-values > 4.46, and *p*-values < 0.001 and Cohen’s *d* values > 1.83).

#### Changes in performance measures with increasing time-on-task

**Fig. 9:**
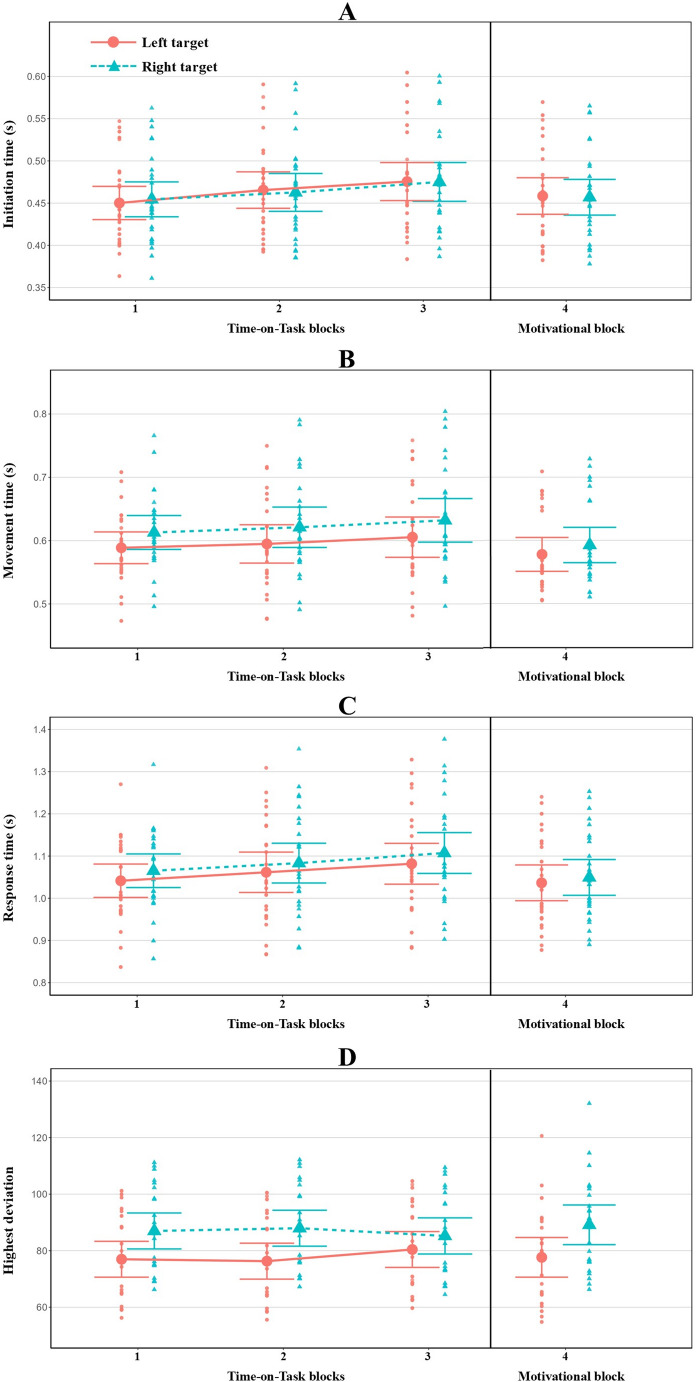
Model-predicted marginal means from LMM analyses addressed to the difference between the two *target presentation sides* (i.e. left vs. right target) for four performance measures (**A**–**D**) in Experiment 2. Error bars represent the 95% confidence intervals. The individual data points represent the predicted within-subject average for each block / trial condition

**Fig. 10:**
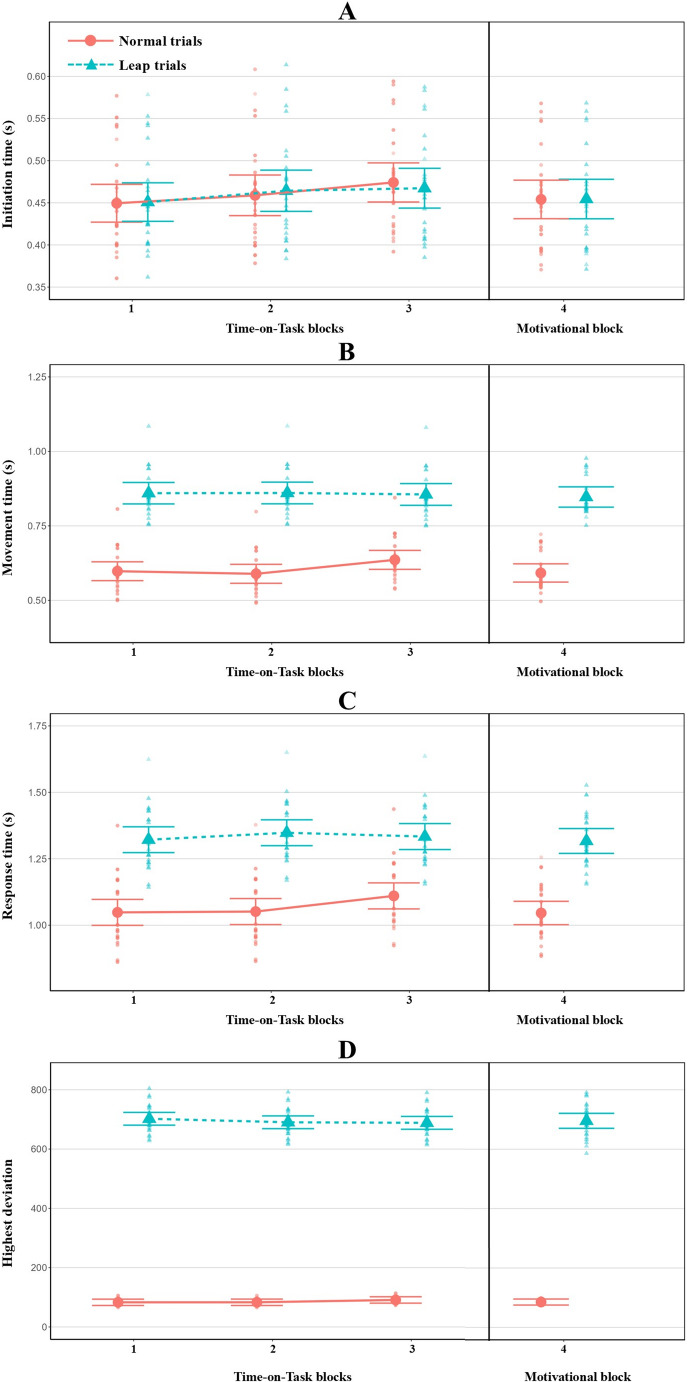
Model-predicted marginal means from LMM analyses addressed to the difference between the *leap and normal trials* for four performance measures (**A**–**D**) in Experiment 2. Error bars represent the 95% confidence intervals. The individual data points represent the within-subject average for each block / trial condition

The results on performance measures in Experiment 2 are presented in Tables 3 and 4, as well as in Figs. 9 and 10. We first report the results of the analyses that involved the comparison of targets presented on the left or right side of the screen. Significant Side main effects were found for MT, RT and HD. Movement execution was slower and more erroneous when the target stimulus was on the right side of the screen. In addition, the analysis of IT and RT revealed significant Block main effects. Post-hoc tests of IT indicated that movement initiation was fastest in the first block, and gradually became slower as a function of time spent on the task (Block 1 vs. Block 2: t(25.9) = -2.68, *p* = .04, Cohen’s *d* = − 0.22; Block 1 vs. Block 3: t(25.8) = -4.42, *p* < .001, Cohen’s *d* = − 0.42; Block 2 vs. Block 3: t(25.7) = -2.96, *p* = .02, Cohen’s *d* = − 0.21). Post-hoc analyses of RT revealed a similar pattern: RT was significantly slower in the third block than in the first (t(25.9) =-3.14, *p* = .01, Cohen’s *d* = − 0.23) and second (t(26.3) =-2.65, *p* = .04, Cohen’s *d* = − 0.12) blocks. None of the Block x Side interactions were significant. Second, the difference between leap and normal trials (i.e. trial types) yielded significant Trial type main effects on MT, RT, and HD confirming that movement execution was longer and more erroneous in leap trials than in normal ones. Significant Block main effects were found for IT and RT. Post-hoc analyses revealed that both IT and RT significantly increased from the first block to the third block (IT: t(26.4) = -2.96, *p* = .02, Cohen’s *d* = -0.39; RT: t(1522) = -3.24, *p* = .004, Cohen’s *d* = − 0.21). The analyses of MT and RT also yielded significant Block x Trial type interactions. Follow-up analyses showed that, in normal trials, but not in leap trials, MT and RT were significantly increased in the third block compared to the first (MT: t(1536) = -2.89, *p* = .02, Cohen’s *d* = − 0.28; RT: t(1527) = -4.39, *p* < .001, Cohen’s *d* = − 0.35) and second (MT: t(1535) = -3.52, *p* = .003, Cohen’s *d* = -0.28; RT: t(1527) = -4.12, *p* < .001, Cohen’s *d* = − 0.33) blocks. In general, ToT mainly seems to have had a detrimental effect in normal trials, whereas the response parameters of leap trials did not seem to be affected by ToT. Similar to Experiment 1, baseline performance was only weakly associated with ToT-related changes (median = − 0.07, range = − 0.21 to 0.36), while the random slopes of Block2 and Block3 showed strong positive correlations (median = 0.86, range = 0.74 to 0.91). For more information, see Supplementary Tables S7 and S8.

#### Motivation-related changes in performance measures

The results of motivation-related changes are summarized in Tables 3 and 4. The analyses of IT, MT and RT yielded significant Block main effects, with all three metrics decreasing significantly in the motivational block compared to the last block of the ToT period. The analyses focusing on the side of targets also showed significant Side main effects for MT, RT, and HD. Movements were slower and more erroneous when they were directed toward the target presented on the right side. Finally, there was a significant Block x Side interaction for HD. Follow-up analyses showed that while there was no significant Side effect in the last block of the ToT (t(3142) = -1.82, *p* = .14, Cohen’s *d* = − 0.09), it was significant in the motivational block (t(3141) = -4.67, *p* < .001, Cohen’s *d* = − 0.23). Movements directed toward the right side turned out to be more erroneous.

The difference between leap and normal trials yielded significant Trial type main effects for MT, RT, and HD. Leap trials elicited longer and more erroneous movement execution than normal ones. The Block main effect was significant in the case of IT and RT, while it was only marginally significant for MT. After the motivational instruction, all three metrics decreased compared to the last block of the ToT. Finally, we also found a significant Block x Trial type interaction for RT, while it was marginally significant for MT. Further analyses showed that both RT and MT significantly decreased in normal trials (RT: t(41.0) = 3.39, *p* = .003, Cohen’s *d* = 0.35; MT: t(47.5) = 2.916, *p* = .01, Cohen’s *d* = 0.26), but they remained unchanged in leap trials (RT: t(77.3) = 0.68, *p* = 1.00, Cohen’s *d* = 0.08; MT: t(109.0) = 0.37, *p* = 1.00, Cohen’s *d* = 0.04). Finally, consistently with Experiment 1, correlations among random-effects suggested stronger motivation-related effects in participants with lower performance in the final block of the ToT phase (median = -0.46, range = -0.88 to -0.23; see Supplementary Table S7 and S8).

**Table 3:** Main effects and interactions by LMM for ToT- and motivation-related changes in *Left and Right target trials* in Experiment 2

	Time-on-Task			Motivation		
	Block (B)	Side (S)	B x S	Block (B)	Side (S)	B x S
*Initiation time*						
df	25.77	25.68	4620.12	25.74	25.40	25.21
F	9.92	0.03	1.73	12.23	0.13	0.05
*p*	< 0.001	0.868	0.177	0.002	0.718	0.830
Marginal **R**^**2**^	0.015			0.012		
Conditional **R**^**2**^	0.527			0.525		
*Movement time*						
df	26.26	25.58	4670.42	26.66	26.43	3120.51
F	1.34	13.87	0.02	14.16	9.06	0.88
*p*	0.278	< 0.001	0.977	< 0.001	0.006	0.347
Marginal **R**^**2**^	0.006			0.011		
Conditional **R**^**2**^	0.161			0.170		
*Response time*						
df	26.13	25.72	4657.44	26.07	3129.77	3129.63
F	5.45	12.87	0.04	18.14	8.83	0.93
*p*	0.011	0.001	0.960	< 0.001	0.003	0.334
Marginal **R**^**2**^	0.009			0.016		
Conditional **R**^**2**^	0.292			0.303		
*Highest deviation*						
df	4705.59	4705.92	4706.42	3140.79	3141.10	3141.22
F	0.14	40.40	2.20	0.15	20.94	3.97
*p*	0.869	< 0.001	0.111	0.702	< 0.001	0.046
Marginal **R**^**2**^	0.009			0.007		
Conditional **R**^**2**^	0.091			0.105		

LMM: linear mixed model; Side: Left vs. Right target; df: denominator df

**Table 4:** Main effects and interactions by LMM for ToT- and motivation-related changes in *Leap and normal trials* in Experiment 2

	Time-on-Task			Motivation		
	Block (B)	Trial type (T)	B x T	Block (B)	Trial type (T)	B x T
*Initiation time*						
df	26.43	1500.64	1497.29	26.14	993.84	994.03
F	4.53	0.00	1.67	8.15	0.78	1.03
*p*	0.020	1.00	0.188	0.008	0.377	0.311
Marginal **R**^**2**^	0.013			0.012		
Conditional **R**^**2**^	0.563			0.528		
*Movement time*						
df	1527.78	25.52	1526.60	25.63	999.23	1000.98
F	2.15	800.15	3.30	3.92	509.17	2.83
*p*	0.116	< 0.001	0.037	0.058	< 0.001	0.093
Marginal **R**^**2**^	0.315			0.299		
Conditional **R**^**2**^	0.419			0.410		
*Response time*						
df	1519.79	24.46	1518.98	26.60	990.67	992.26
F	5.30	699.82	5.30	5.46	493.11	4.34
*p*	0.005	< 0.001	0.005	0.027	< 0.001	0.038
Marginal **R**^**2**^	0.273			0.264		
Conditional **R**^**2**^	0.488			0.478		
*Highest deviation*						
df	1540.81	26.04	1540.67	30.28	25.97	999.36
F	0.59	3564.05	1.99	0.00	2568.99	1.11
*p*	0.554	< 0.001	0.137	0.962	< 0.001	0.293
Marginal **R**^**2**^	0.913			0.903		
Conditional **R**^**2**^	0.924			0.918		

LMM: linear mixed model; Trial type: Leap vs. Normal trials; df: denominator df

### Discussion

ToT was accompanied by increased subjective fatigue and perceived mental workload. Performance data from Experiment 2 were analysed using two approaches: (1) analysis of the effect of target presentation sides; and (2) analysis of the difference between leap and normal trials. The results of both analyses suggested that the performance of the pointing task deteriorated with increasing ToT.

Similarly to Experiment 1, a significant effect of the target side was observed in this experiment with participants executing movements to the left side faster. In contrast to Experiment 1, however, the benefit of left targets remained unchanged with ToT, which can be explained by a relatively low demand on cognitive control required by the task in Experiment 2. More specifically, the majority of the trials (i.e. normal trials) in Experiment 2 were simple pointing trials without any response conflict, or any other cognitively demanding condition. This demand on cognitive control in normal trials was possibly not high enough to induce a stronger rightward attentional bias with ToT; the left hemisphere-lateralized attentional process did not take the dominance over the right hemispheric process.

In the leap trials we found that the execution of movements was slower and less accurate than in normal trials. This compromised performance was predictable, because participants had to modify the path of a movement they had already started, which obviously resulted in a slower movement along with a longer path. The movement towards the target with the altered position was plausibly preceded by at least two cognitive operations. First, participants were required to recognize that the movement initiated based on the original movement plan was no longer appropriate, conflicting with the plan on which the movement towards the new target should be based. Second, as another important cognitive operation, the execution of the actual movement directed toward the original target location had to be inhibited and then redirected towards the new target. The movement during leap trials was thus cognitively complex and therefore plausibly required a high level of cognitive control. On the basis of this cognitive complexity, a fatigue-induced deterioration was assumed; however, contrary to this hypothesis, performance in the leap trials did not change with ToT. This finding is particularly interesting in light of the fact that performance in normal trials did significantly suffer due to ToT. One possible explanation for the differential fatigue sensitivity of the leap and normal trials is the difference between phasic and tonic vigilance. While in leap trials, the cognitive flexibility required a rapid increase in the phasic vigilance level, in normal trials, the level of tonic vigilance may have been the dominant performance modulatory factor. Previous studies have found that the rapid phasic changes in spatial orientation (Matuz et al., 2022a, b), as well as responses to infrequent stimuli (i.e. oddball stimuli; Takács et al., 2019), are resistant to enhanced fatigue. This fits with the lack of effects of fatigue in the leap trials. In contrast, the monotonic performance in normal trials may have required a sustained tonic vigilance level that may be more difficult to uphold with increasing ToT. Finally, similarly to the first experiment, in the second experiment we also found evidence of a link between motivation and fatigue. Following the motivational message, performance in the normal trials improved, indicating that motivating participants to perform better reduced fatigue-induced task disengagement.

## General discussion

Two experiments addressed the question of whether the performance of simple pointing movements is sensitive to fatigue induced by prolonged task performance (i.e. time-on-task). A fatigue-related effect on movements was hypothesized based on previous studies showing that both the planning and executive phases of movements require cognitive control, which is known to be compromised when individuals’ fatigue level is raised (Janczyk & Kunde, 2010; Liu et al., 2008; Matuz et al., 2022a, b). The two experiments focused on ToT-related changes in resolving response conflict as a specific aspect of cognitive control. In the first experiment, the response conflict was induced by the incongruence in the flanker cues, while in the second experiment, it was the unexpected change in target locations.

The first experiment shows that the incongruency effect is robust and affected not only the movement planning phase but also movement execution. Nevertheless, contrary to our hypothesis, with increasing ToT, participants did not show a general decline in performance of the incongruent trials. Performance in incongruent trials only deteriorated with ToT when their execution required leftward movements (i.e. when target stimuli appeared in the left visual field). As noted earlier (see Discussion of Experiment 1), two factors may underlie this finding. First, the response selection process, which also affects the movement execution phase, increased the cognitive control demands of movement execution; that is, cognitive control demands during the execution phase of incongruent trials were high (i.e. higher than expected; see the conceptual framework illustrated in Fig. 1), thereby increasing the fatigue sensitivity of movement execution. Second, performance deterioration required movement execution to occur in the left visual field, where attentional processing likely declined as a consequence of ToT.

This latter conclusion is supported by the finding that movement initiation showed a greater decrease for leftward movements than for rightward movements over ToT. This suggests an attentional benefit of the right visual field under fatigue, which is in line with previous studies that also observed a rightward spatial attentional shift with reduced alertness or vigilance. For example, Manly et al. (2005) induced fatigue with a landmark test (i.e. line section task) performed for more than one hour and found evidence that while a relatively high alertness level was associated with a leftward attentional orientation, a decrease in alertness levels resulted in a rightward attentional shift. Benwell et al. (2013) reported similar results in using a bisection task, but they observed that the attentional shift due to fatigue also depended on the difficulty of the task. A rightward bias with ToT was only observed for trials with higher cognitive demands (i.e. longer lines). In addition, Paladini et al. (2016) demonstrated that with increasing ToT, individuals seem to have a lower shift cost (from the central fixation to the peripheral target) for right than for left targets. This is in line with the finding of the first experiment, i.e. that the incongruent trials that caused response conflict were the only ones in which the spatial attentional orientation shifted to the right with increasing ToT.

Similarly to the current study, the role of task disengagement under fatigue is often investigated by examining the effect of manipulation of task-related motivation (see, for example, Hopstaken et al., 2015a, b, 2016). Such manipulation is often found to reduce the decline in performance caused by prolonged cognitive performance. In line with this, the motivational message used in the first experiment improved performance in the subsequent pointing trials. This finding further supports the concept that task disengagement is a critical underlying cause of performance deterioration due to mental fatigue. The present experiments reinforce this concept through the results on a task (i.e. visually guided pointing task) that has not been previously investigated from a fatigue-motivation perspective. However, the results in the motivational block also showed that, compared to the easier conditions, the increase in participants’ motivation did not compensate for the ToT-related deterioration in more demanding trial conditions after the motivational manipulation, and the improvement in pointing movements did not mitigate the disadvantage of incongruent trials with leftward movements. These findings suggest that, although more attentional resources were directed toward the task after the motivational message resulting in faster pointing movements overall, this did not compensate for the fatigue-related decline in trials requiring a high level of cognitive control (i.e. incongruent trials with leftward movements).

An alternative explanation for the improvement in performance observed after the motivational message is that the short break during the presentation of the message, rather than the content of the message, had a positive effect on performance. This question is particularly relevant because the way we analysed the effect of the motivational message – by comparing performance in the block preceding and the block following the message – is similar to what Schumann et al. (2022) identified as a method for computing local rest-break effects. This is a pre-post comparison approach used to capture the local benefit of a break; however, based on previous studies, it seems unlikely that the observed effect in the present study reflects a mere break effect rather than a motivational effect. Although breaks during ToT periods often have beneficial impacts, improvements in objective performance are typically associated with longer breaks. In a systematic review, Albulescu et al. (2022) found that breaks shorter than 10 min primarily reduce subjective fatigue and are unlikely to improve subsequent objective cognitive performance. In the present study, the motivational message was displayed for only 20 s, making it unlikely that the break alone accounted for the observed performance improvement. Nevertheless, a limitation of the study is that the motivational message was not more specific; that is, the message did not specifically indicate the degree of performance improvement rewarded by a shorter task period. The non-specific message form was chosen because a more specific message would likely influence the speed and accuracy of pointing movements unequally, thereby amplifying a speed–accuracy trade-off. To more precisely disentangle the motivational effect from a potential break effect, further studies will be required that compare the effects of multiple types of breaks and motivational messages.

In the second experiment, the infrequent leap trials created a specific type of response conflict in which the initiated movement did not match the path required to the new target. Resolving the conflict between the already initiated movement and the newly required direction needed cognitive flexibility. In numerous previous studies, mental fatigue has been found to lead to greater behavioural rigidity (e.g. Matuz et al., 2019; van der Linden et al., 2003), and these previous findings led to our hypothesis of fatigue-related lowered performance in the leap trials of the second experiment. This hypothesis, however, was not supported by the results. Contrary to expectations, the infrequent leap trials caused the same level of difficulty for participants at the beginning of the ToT period as at the end, despite their increasing subjective feelings of fatigue. In addition, leap trial performance did not change after the motivational manipulation either. A possible explanation for the lack of fatigue effect in leap trials is that adjusting the initiated movement path toward the new, unexpected target required a rapid, phasic vigilance increase in spatial attention. However, as shown in a previous study with a pointing task using spatial cues, rapid shifts in spatial attention seem to be relatively resistant to increased fatigue (Matuz et al., 2022a, b). Not phasic but sustained vigilance may, however, have affected the normal trials of the second experiment. The performance of these trials with a relatively low cognitive demand worsened under ToT, but it improved again after the motivation manipulation. The frequency of normal trials may particularly have been a challenge for sustained attention, which possibly became compromised due to a decline in sustained (tonic) vigilance turning to a compromised performance of normal trials.

As a limitation of our study, we must acknowledge that given the large number of models tested the risk of false positives is inherently higher when multiple analyses are conducted, especially when sample size is limited. However, random-effects model reduction was performed only in cases where singular fit warnings were encountered, and no tests (e.g., *p*-values) were examined to guide model simplification. This approach ensured that reductions were made purely for convergence purposes and not to selectively favour any effects. While this strategy preserves the validity of the fixed-effect estimates, the relatively large number of models remains a limitation, and results – particularly small or marginal effects – should be interpreted with caution. In addition, future research should aim to develop a better structured and mathematically more tractable model to precisely characterize the mechanisms linking fatigue, cognitive control, and movement performance.

## Conclusion

In conclusion, across two experiments we demonstrated that performance in visually guided pointing tasks tends to decrease due to ToT-induced mental fatigue. More specifically, we found that ToT reliably impaired movement performance, particularly by slowing movement initiation, as fatigued people seem to take longer to prepare and initiate their movement. Specifically, Experiment 1 shows that fatigue mainly seems to negatively affect pointing performance during control-demanding trials when it is required to initiate a leftward movement and to inhibit the influence of distracting cues (i.e. leftward movement trials with incongruent flankers). This finding is consistent with a fatigue-related rightward attentional bias and suggests that sustained cognitive control under conflicting information becomes increasingly difficult with prolonged task performance. These results are in line with previous studies highlighting the fatigue sensitivity of attentional selection (Faber et al., 2012a, 2012b; Tanaka et al., 2012; Csathó et al., 2012; Möckel et al., 2015). The present findings also extend this line of research by demonstrating that the vulnerability of selective attention to fatigue may also be manifested in the domain of movement control.

However, the results of Experiment 2 indicate that the level of cognitive control required (low vs. high) does not necessarily determine the extent of performance deterioration. In contrast to Experiment 1, Experiment 2 revealed that performance in rare, cognitively demanding leap trials – requiring rapid movement replanning and cognitive flexibility – was largely resistant to fatigue. Instead, fatigue predominantly affected frequent, low-demand trials, indicating a decline in tonic vigilance rather than phasic control mechanisms. This finding seems to point to the relevance of the arousal-eliciting nature of events in relation to mental fatigue. Events (normal trials in our case) that are relatively regular and require tonic vigilance, seem to be more difficult to perform when one becomes fatigued. Rare events (e.g. leap trials in Experiment 2), which are likely to be associated with increased phasic vigilance, appear to be relatively robust to the effects of fatigue.

Importantly, across both experiments, motivational manipulations effectively mitigated fatigue-related performance decrements, highlighting the compensatory role of task engagement and supporting the motivational concept of mental fatigue. However, motivation did not fully offset impairments in the most cognitively demanding conditions (i.e. the detrimental effect of incongruence on movements in Experiment 1), suggesting there are limits to compensation when control demands are high.

Taken together, these findings emphasize that mental fatigue does not uniformly degrade motor performance but selectively impacts movement planning and execution depending on attentional demands and conflict processing. By integrating cognitive control and motivation within a movement-based paradigm, the present study supports and extends current models of mental fatigue.

## Supplementary Information

Below is the link to the electronic supplementary material.

Supplementary Material 1

## Data Availability

The dataset, along with the code to run the experiments, is available on the Open Science Framework: https://osf.io/6evqs/?view_only=5e4741280d3e402d8e5361c93484dc35.
