# Supplementary material for: Response conflicts in visually guided movements under mental fatigue
Source: Psychol Res. 2026 Apr 24;90(3):79. doi: 10.1007/s00426-026-02293-7 (PMC13109180; doi:10.1007/s00426-026-02293-7)
Supplement: Supplementary file 1 — Supplementary Material 1 [file 426_2026_2293_MOESM1_ESM.docx]

**Supplementary material**

**Response conflicts in visually-guided movements under mental fatigue**

**
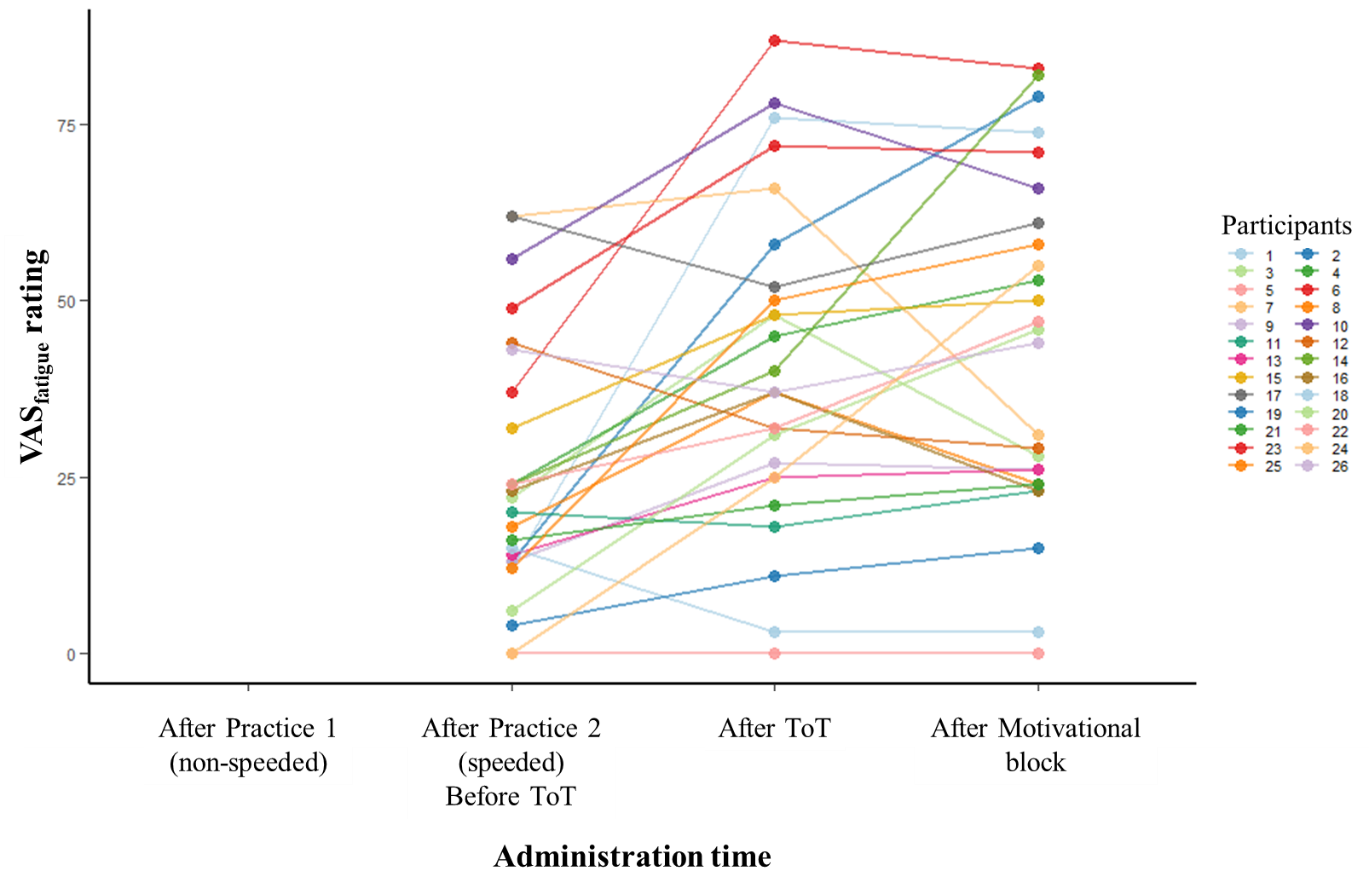
**

**Figure S1.** Individual VAS_fatigue_ (subjective fatigue) data in Experiment 1. The blank data column indicates that VAS_fatigue_ was not administered after Practice 1. ToT: Time-on-Task.

**
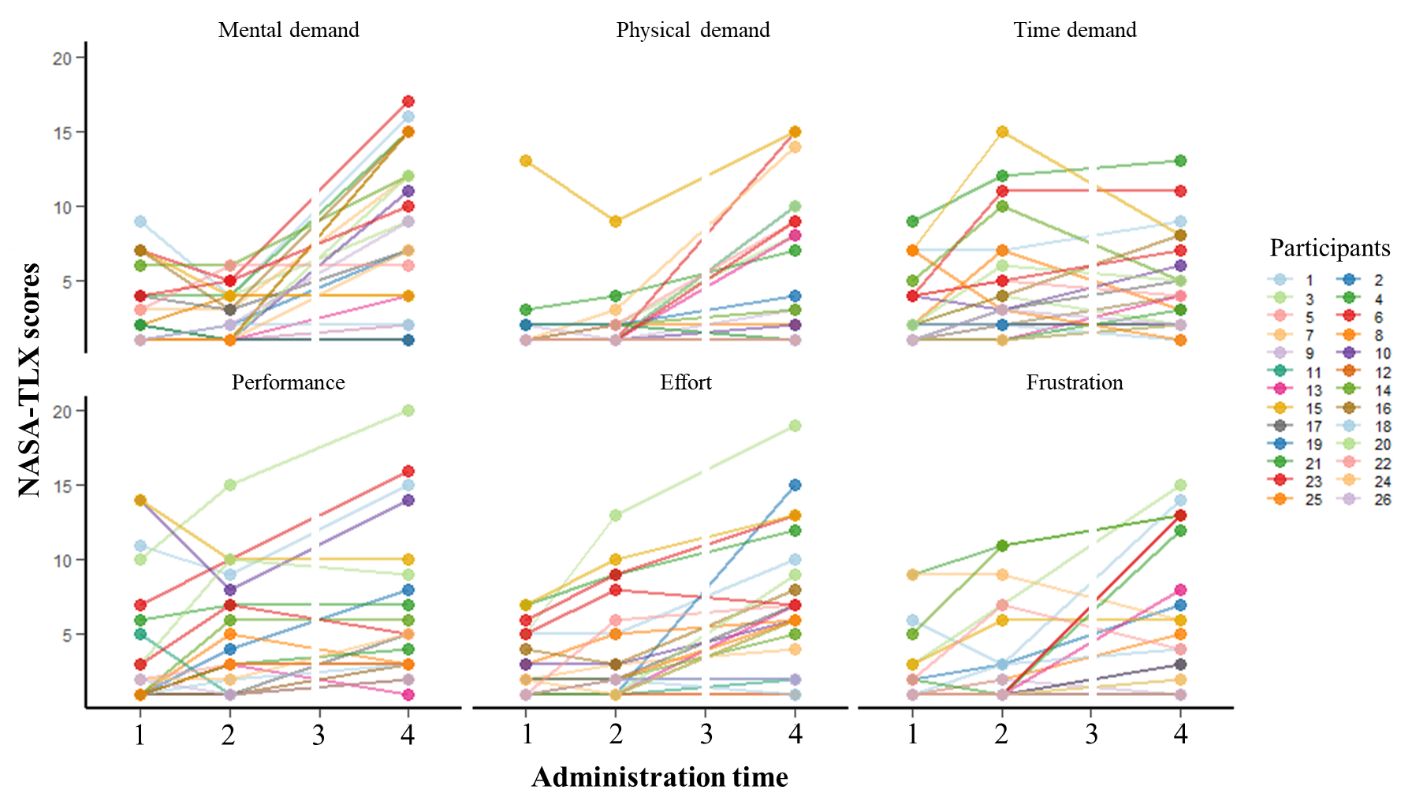
**

**Figure S2.** Individual NASA-TLX (perceived workload) scores in Experiment 1. The blank data column (i.e. Administration time: 3) indicates that NASA-TLX was not administered after Time-on-Task. 1: After Practice 1 (non-speeded); 2: After Practice 2 (speeded), Before Time-on-Task; 3: After Time-on-Task; 4: After Motivational block.

**
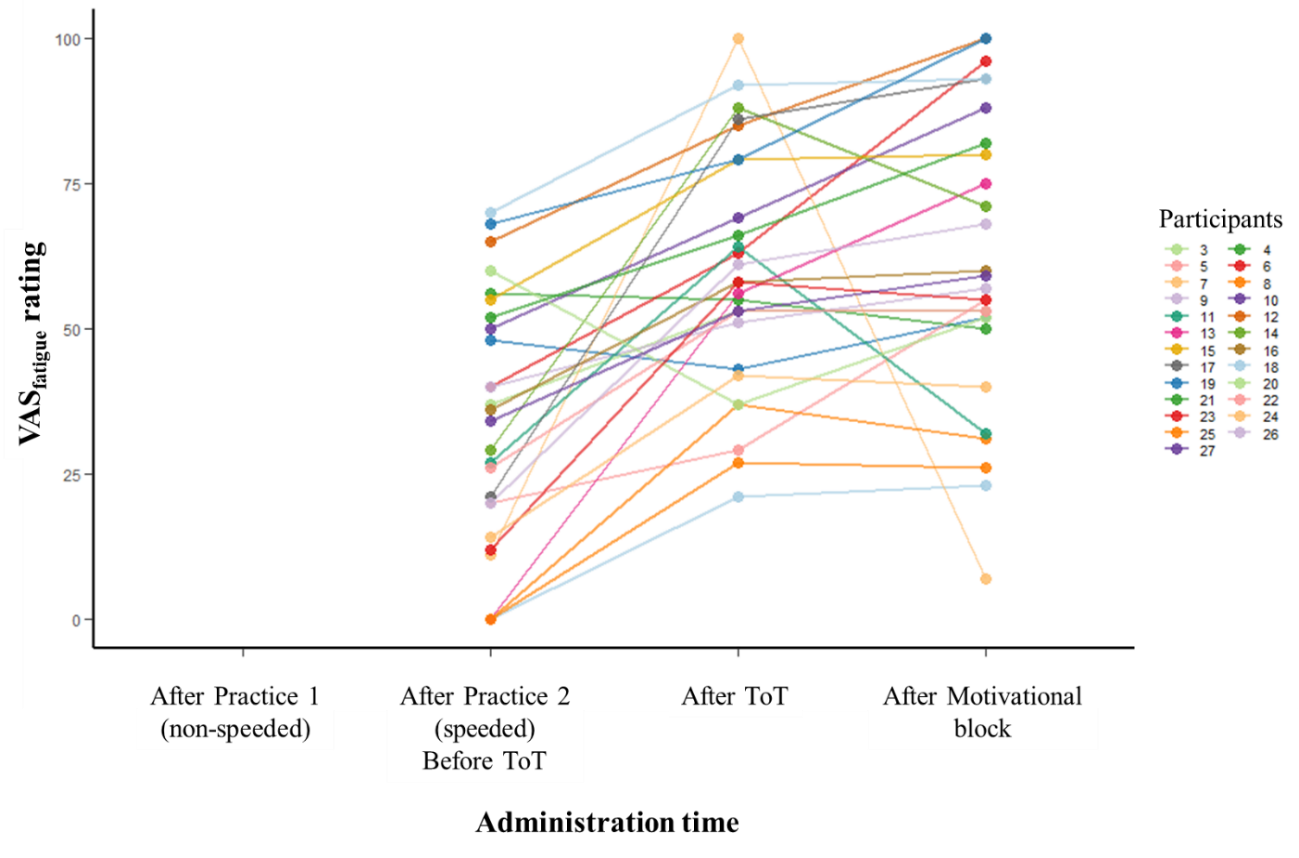
Figure S3.** Individual VAS_fatigue_ (subjective fatigue) ratings in Experiment 2. The blank data column indicates that VAS_fatigue_ was not administered after Practice 1. ToT: Time-on-Task.

**
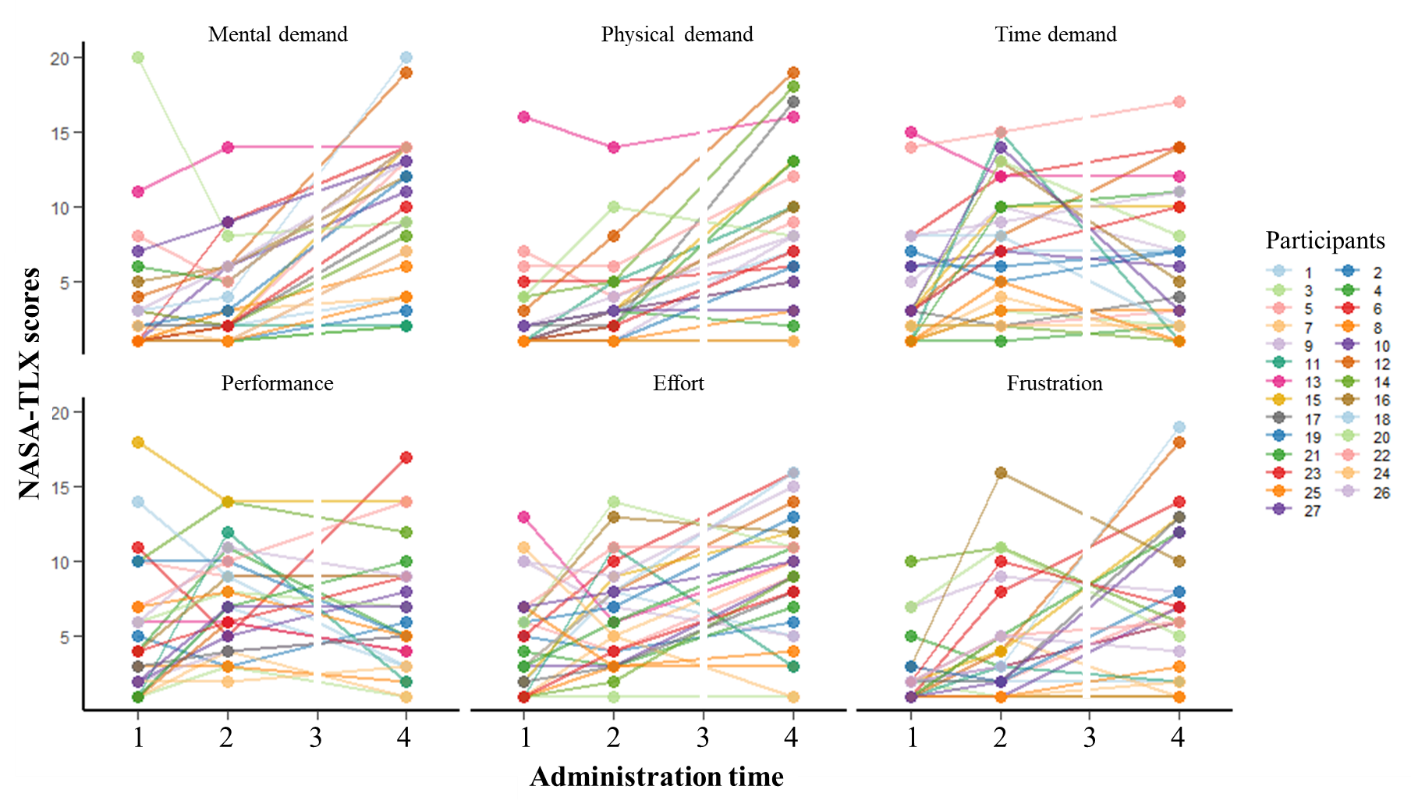
Figure S4.** Individual NASA-TLX scores (perceived workload) in Experiment 2. The blank data column (i.e. Administration time: 3) indicates that NASA-TLX was not administered after Time-on-Task. 1: After Practice 1 (non-speeded); 2: After Practice 2 (speeded), Before Time-on-Task; 3: After Time-on-Task; 4: After Motivational block.

**
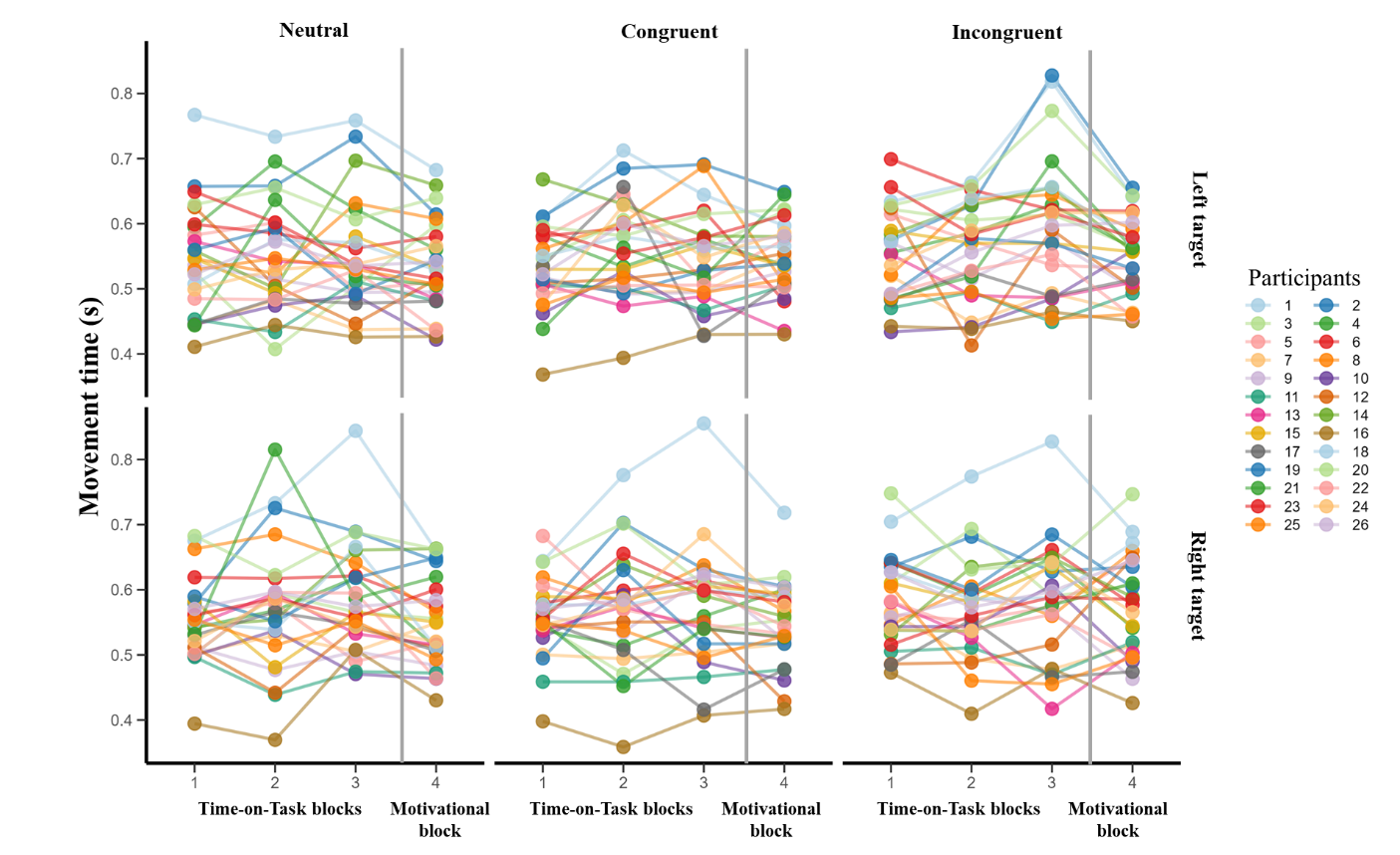

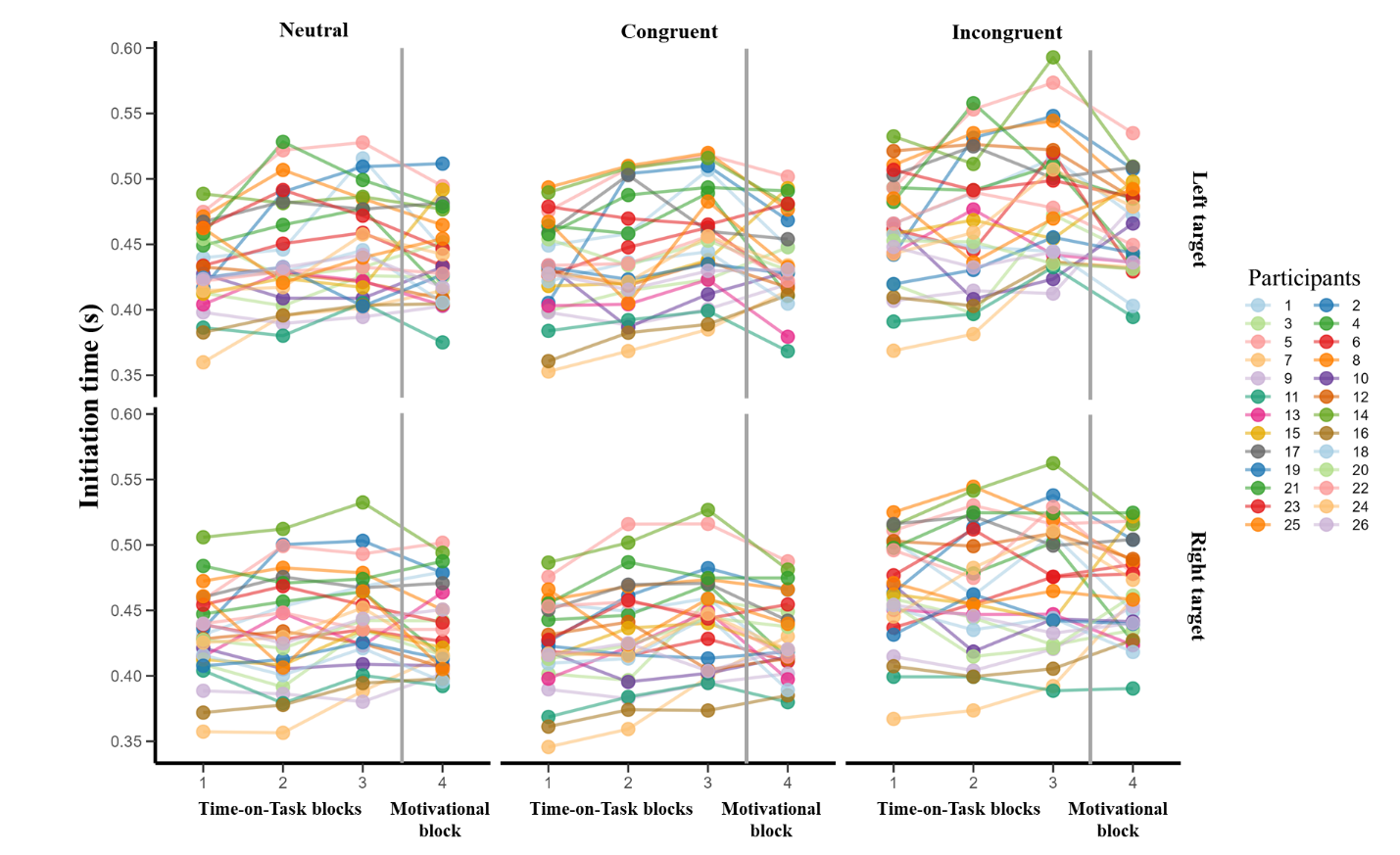
Figure S5.** Individual initiation time data in experiment 1 separately for each trial condition in each block of trials.

**Figure S6.** Individual movement time data in experiment 1 separately for each trial condition in each block of trials.

**
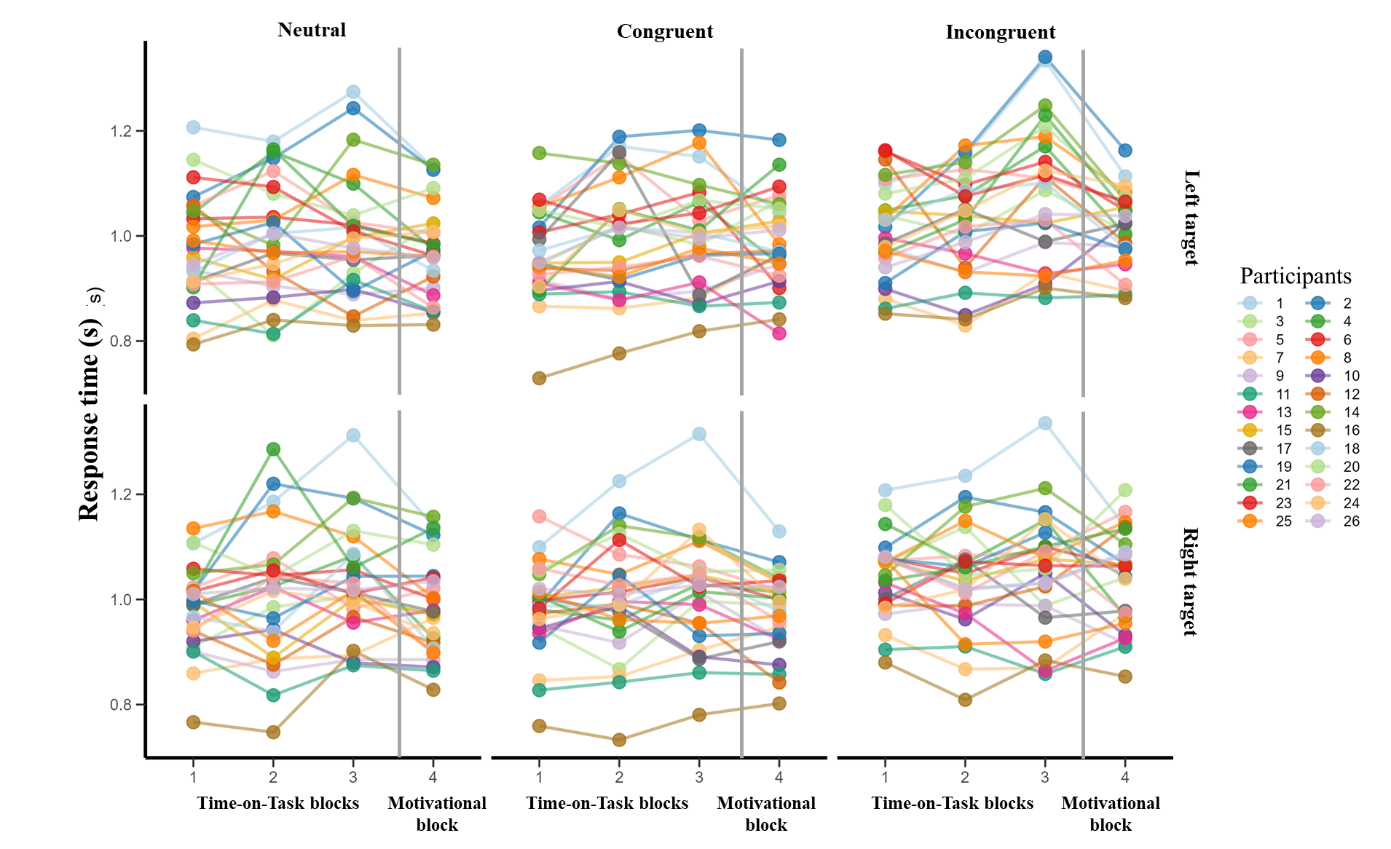
Figure S7.** Individual response time data in experiment 1 separately for each trial condition in each block of trials.

**
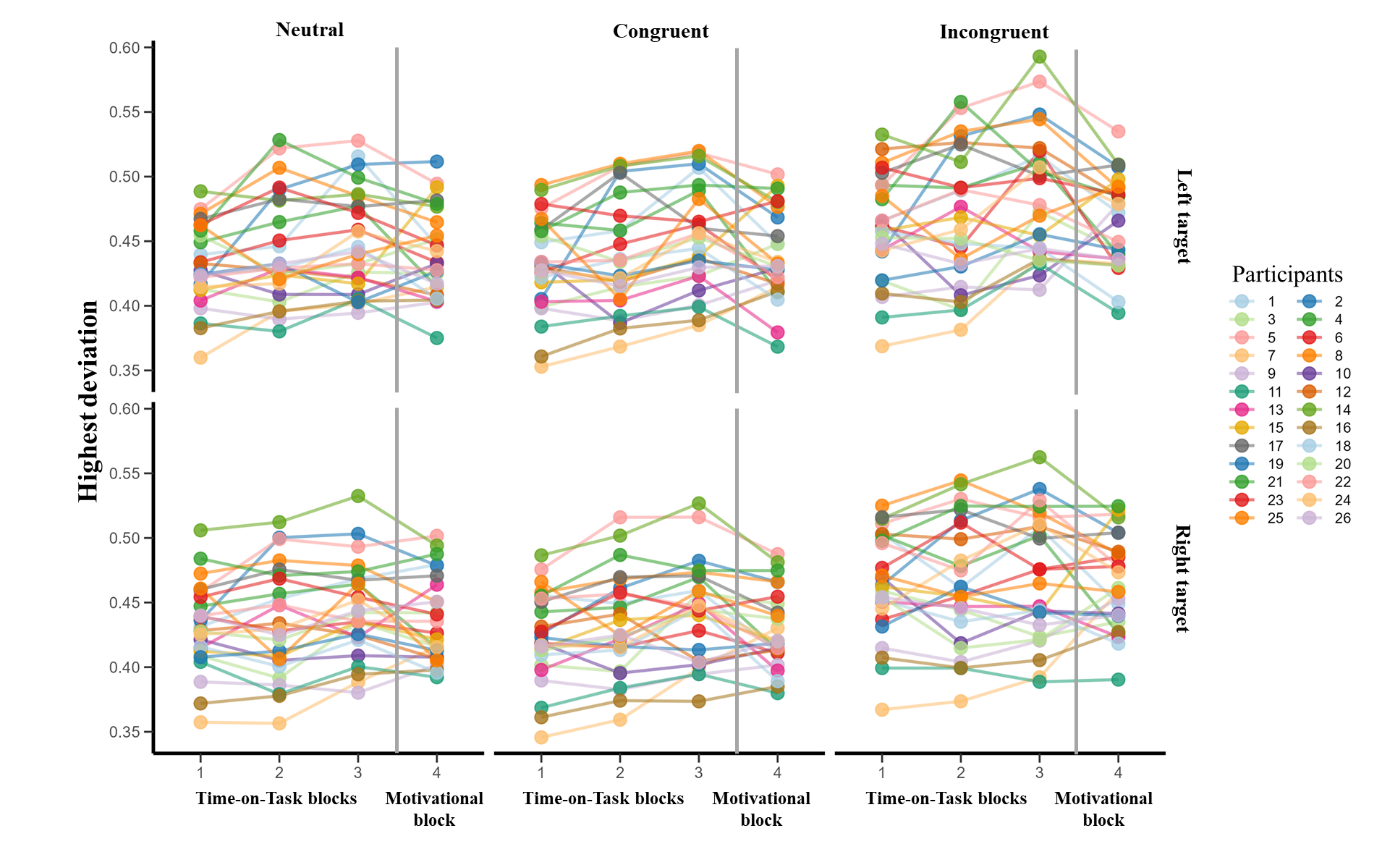
**

**Figure S8.** Individual highest deviation data in experiment 1 separately for each trial condition in each block of trials.

**
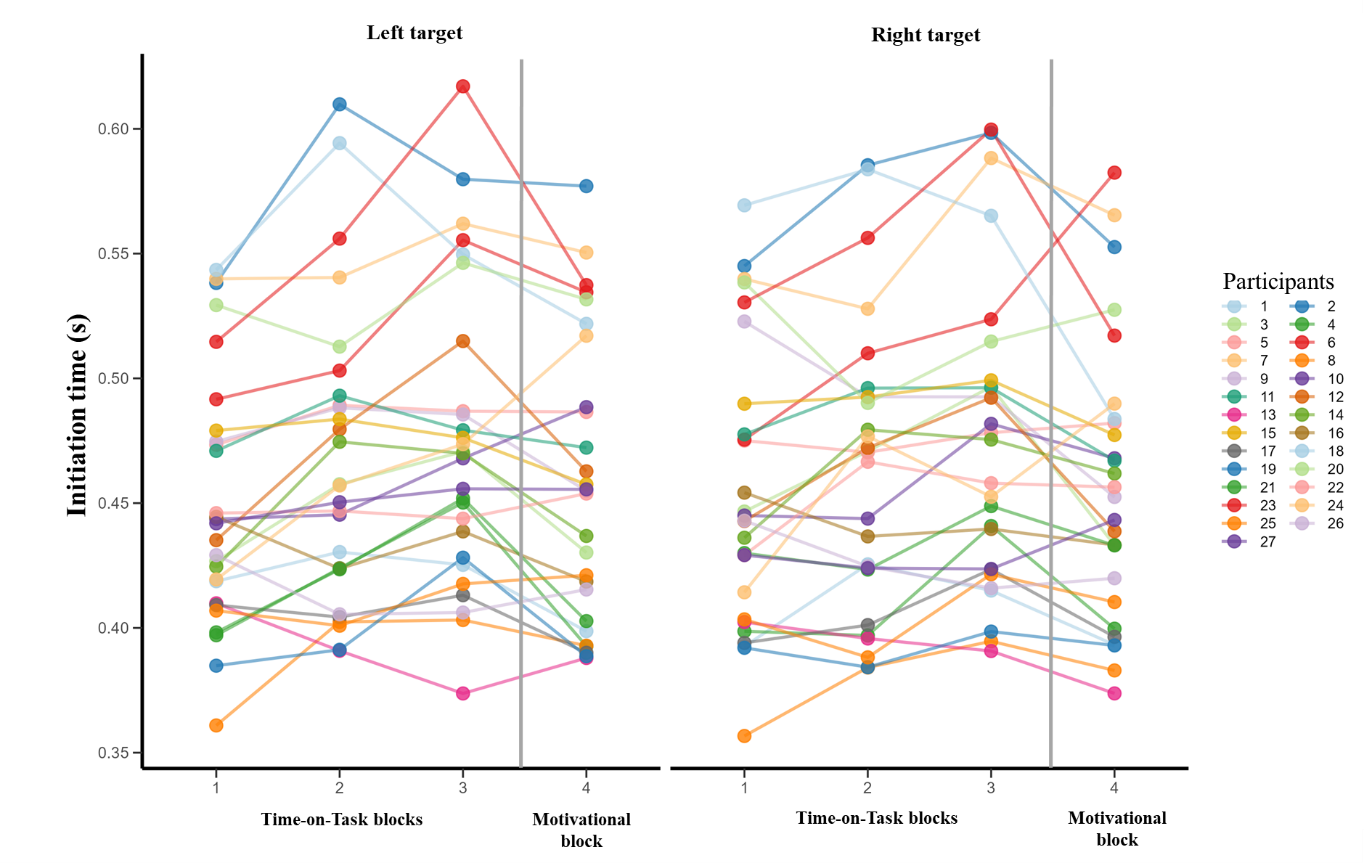
**

**Figure S9.** Individual initiation time data in experiment 2 separately for left and right target position in each block of trials.

**
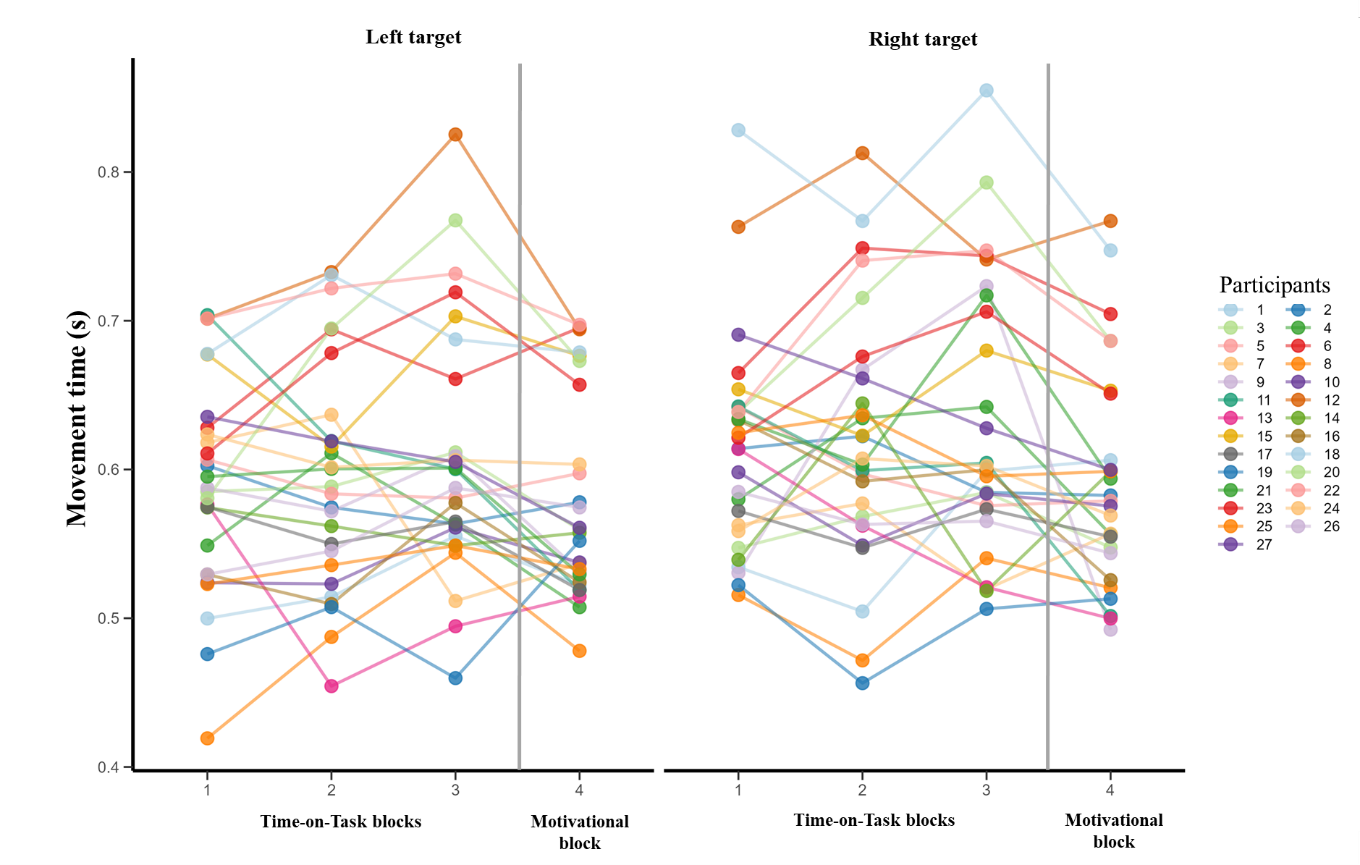
**

**Figure S10.** Individual movement time data in experiment 2 separately for left and right target position in each block of trials.

**
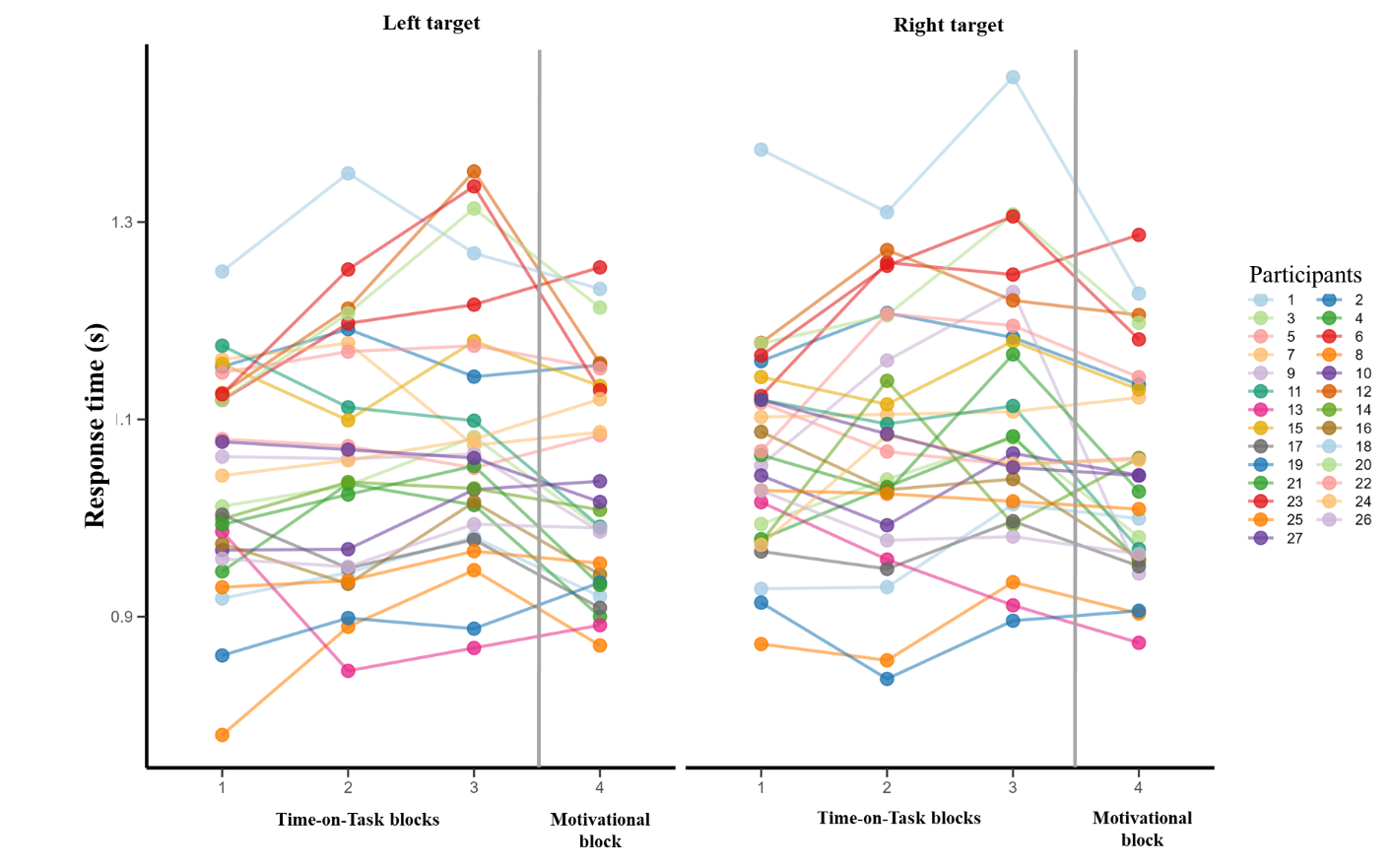
**

**Figure S11.** Individual response time data in experiment 2 separately for left and right target position in each block of trials.

**
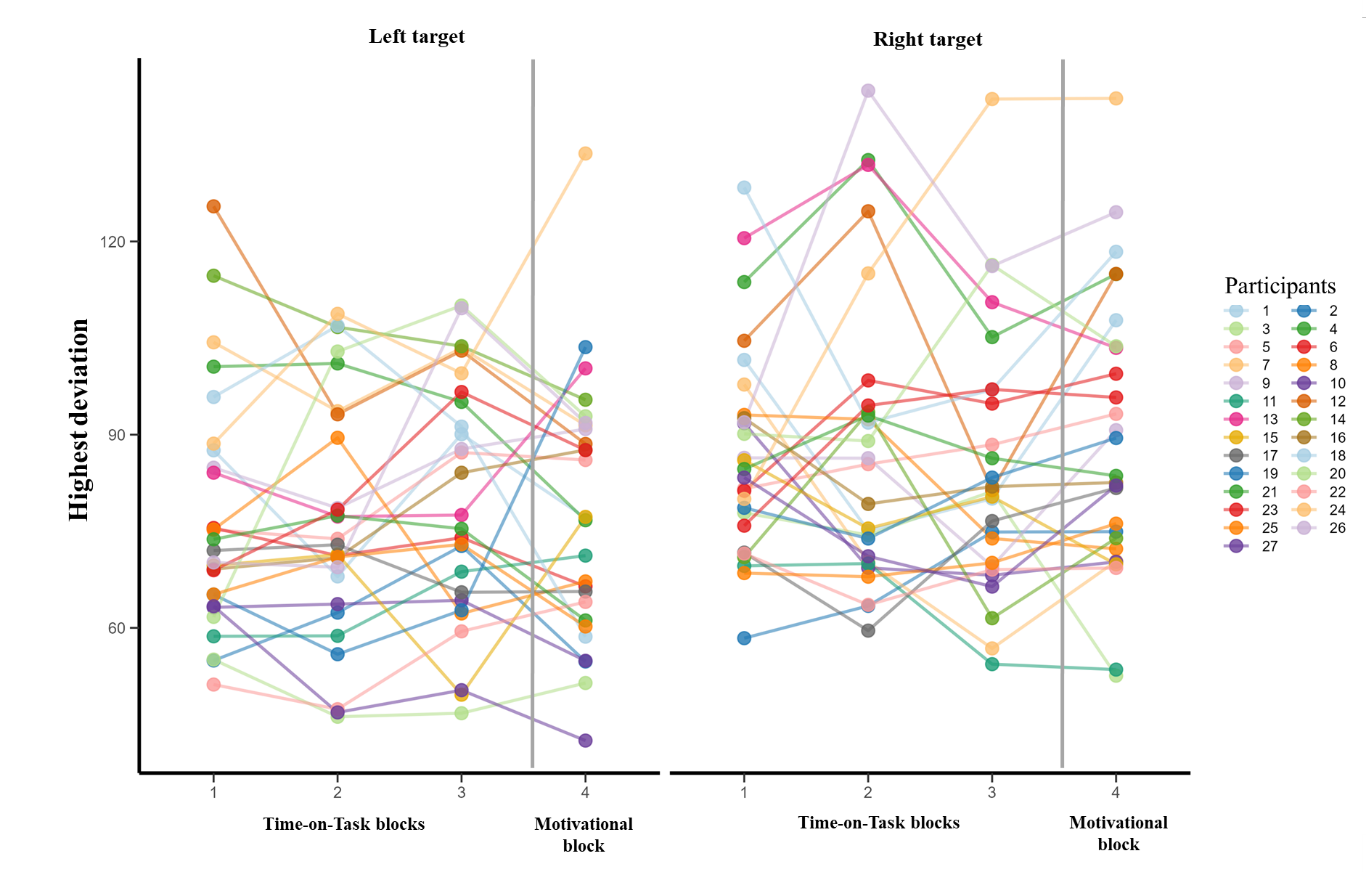
**

**Figure S12.** Individual highest deviation data in experiment 2 separately for left and right target position in each block of trials.


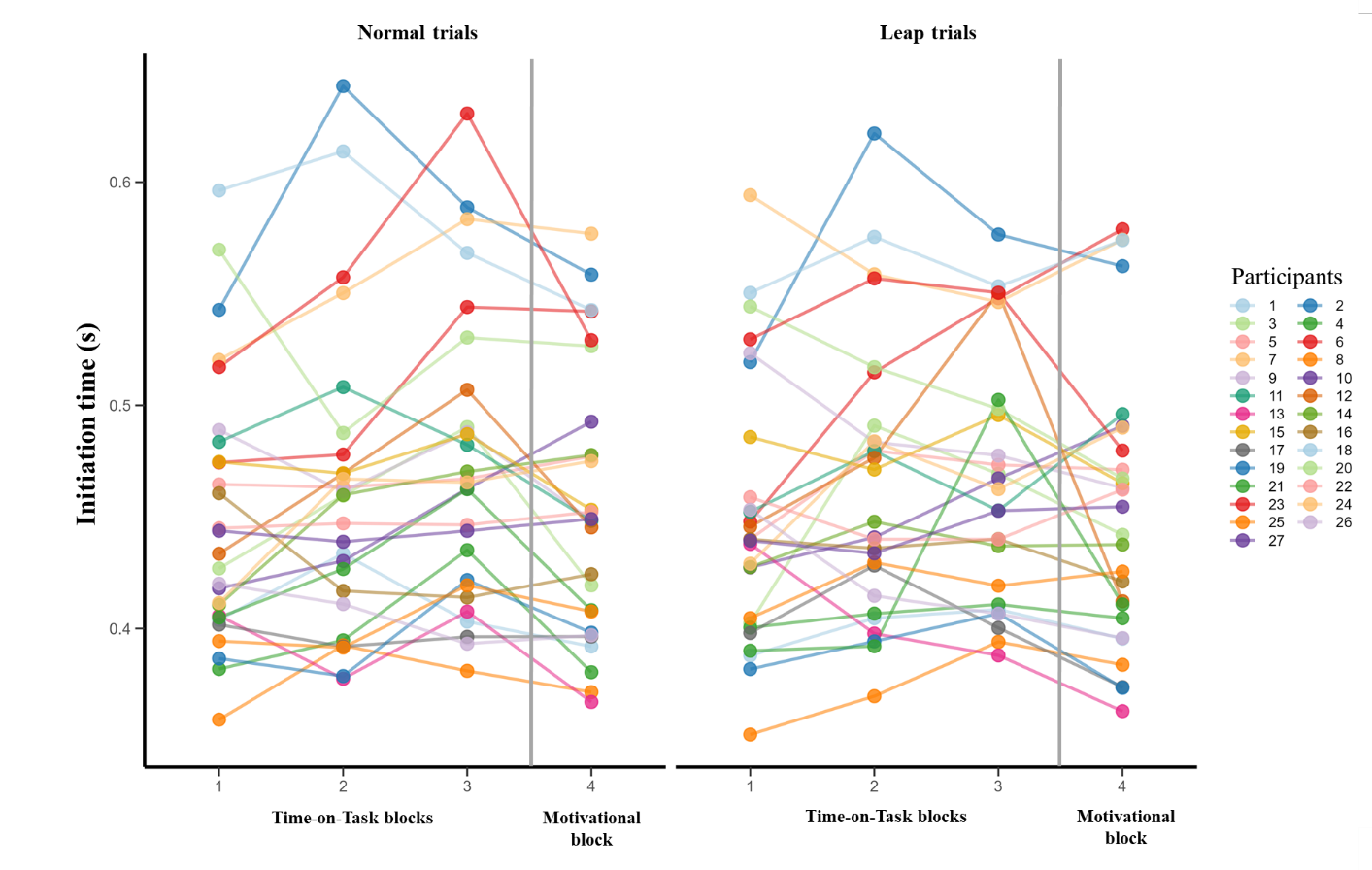


**Figure S13.** Individual initiation time data in experiment 2 separately for normal and leap trial conditions in each block of trials.

**
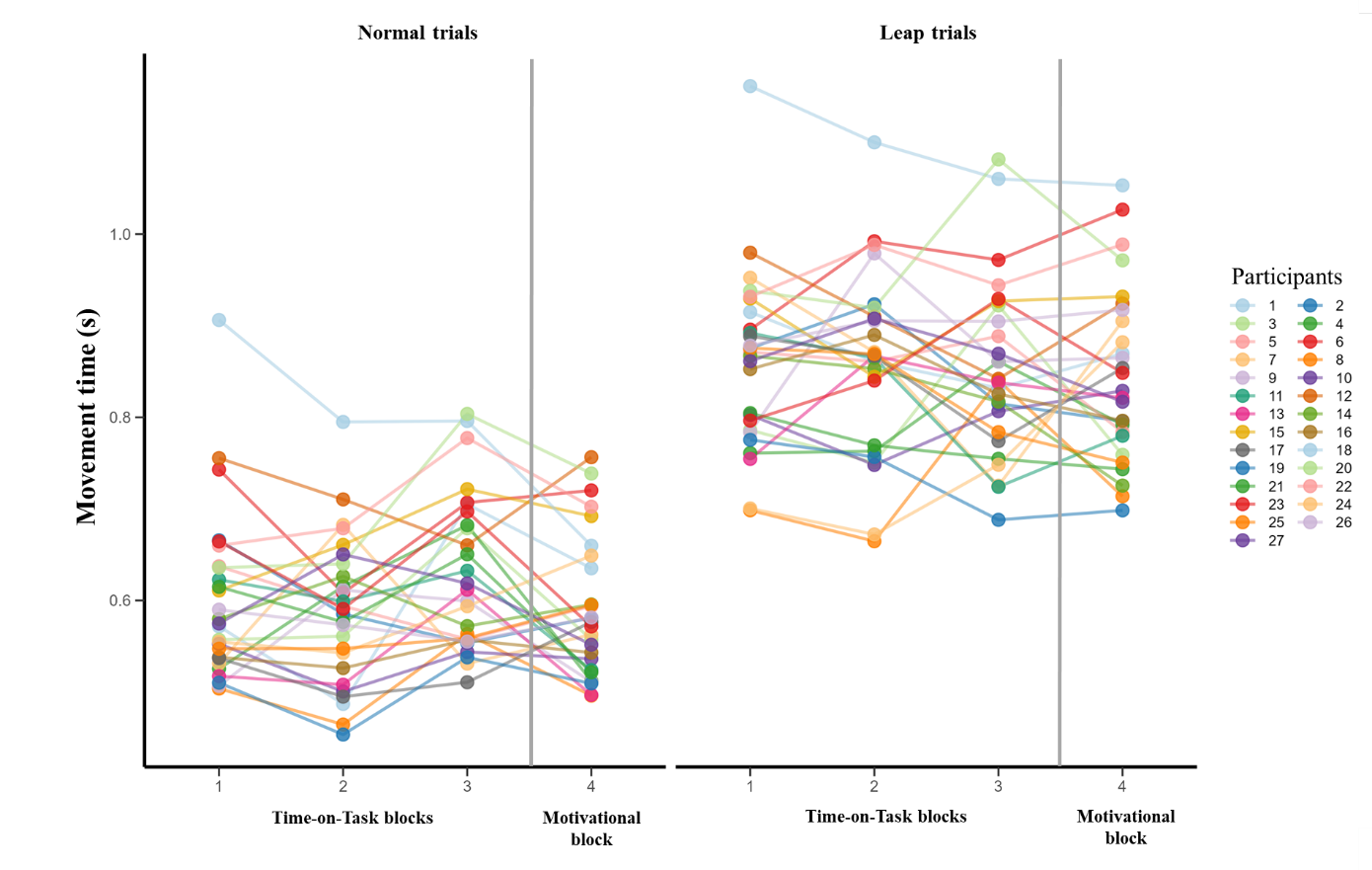
**

**Figure S14.** Individual movement time data in experiment 2 separately for normal and leap trial conditions in each block of trials.


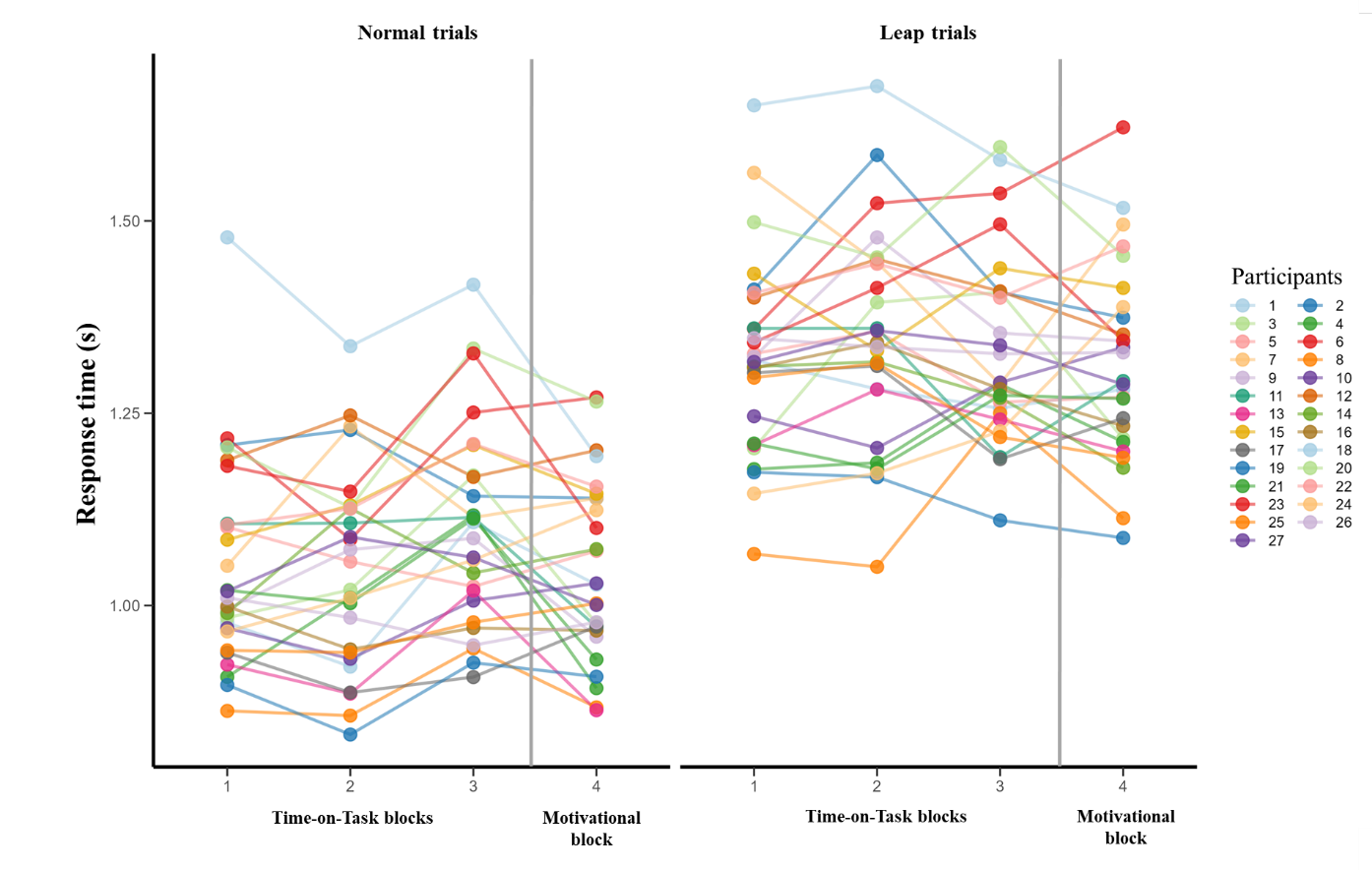


**Figure S15.** Individual response time data in experiment 2 separately for normal and leap trial conditions in each block of trials.

**
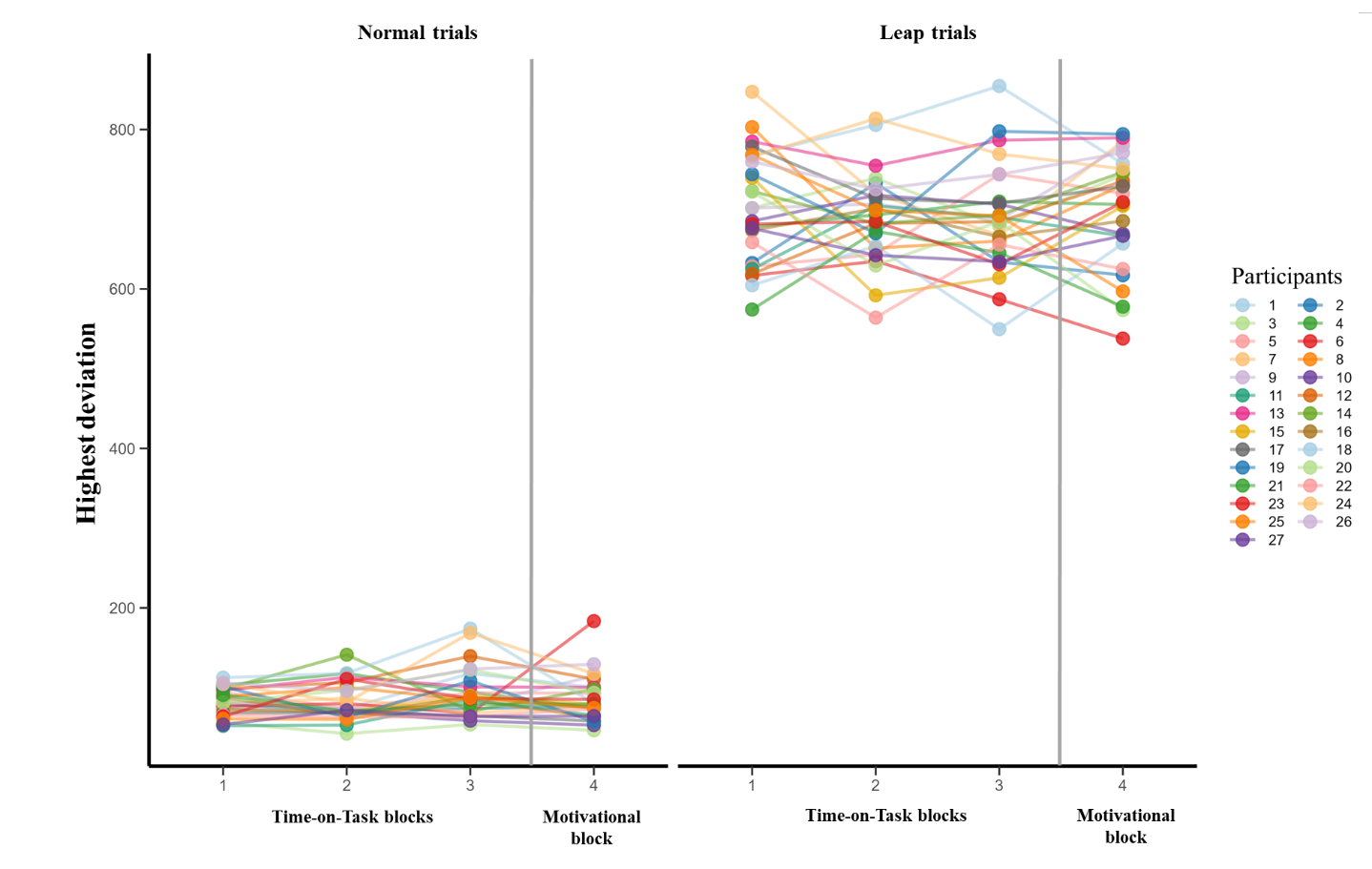
**

**Figure S16.** Individual highest deviation data in experiment 2 separately for normal and leap trial conditions in each block of trials.

**
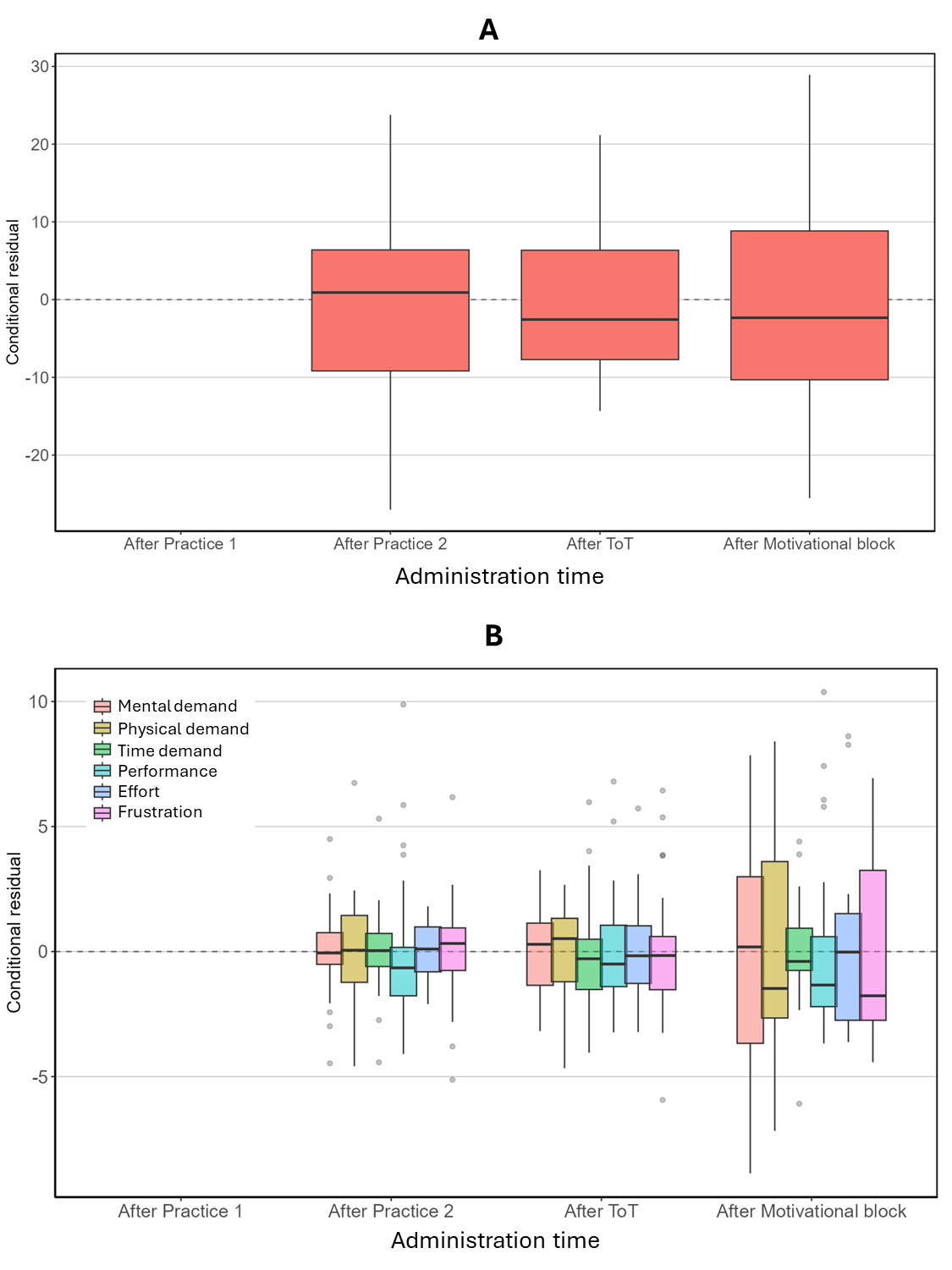
**

**Figure S17.** Conditional residual plots from LMM analyses for subjective fatigue (A) and perceived workload (B) in Experiment 1.


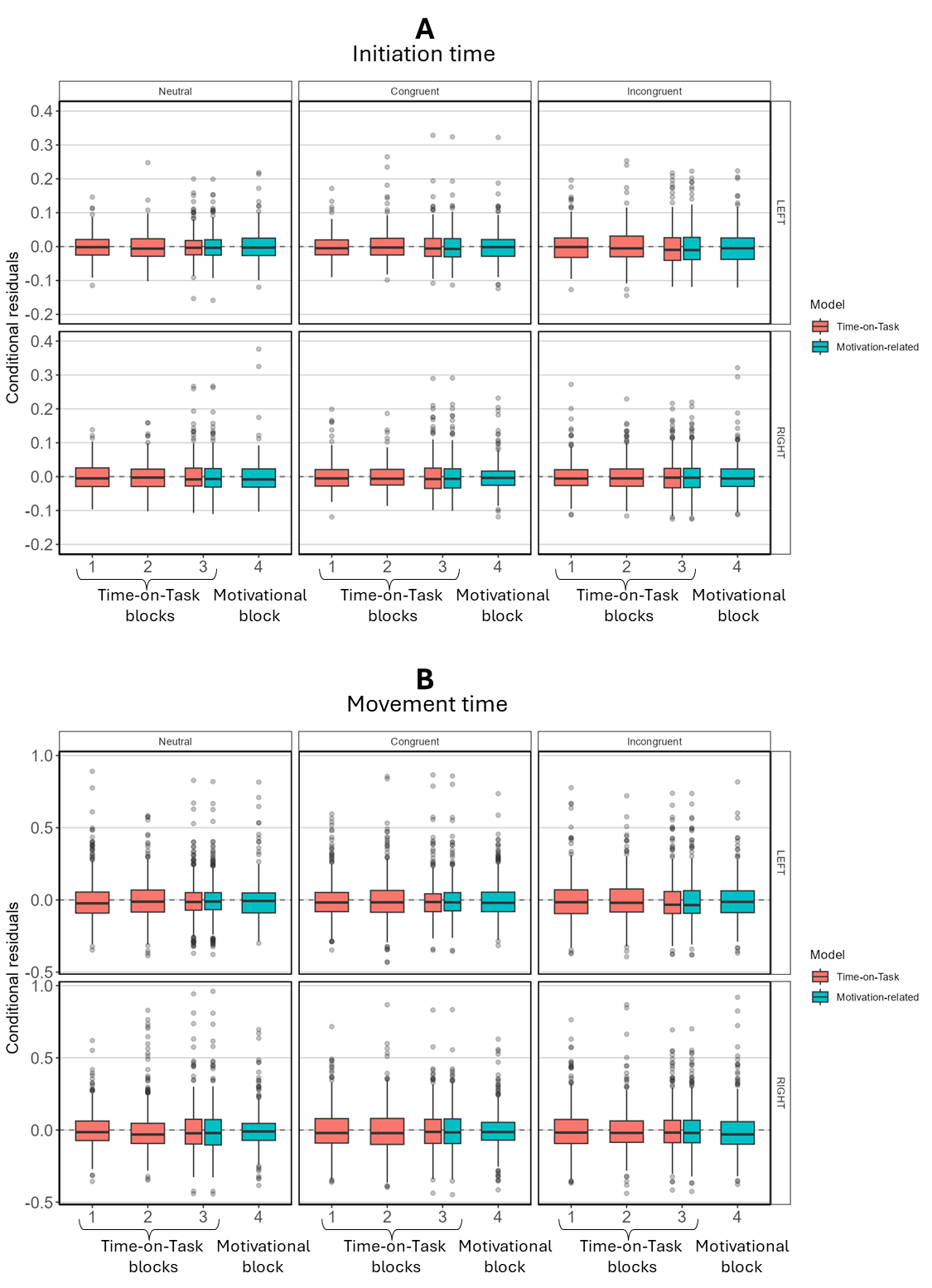


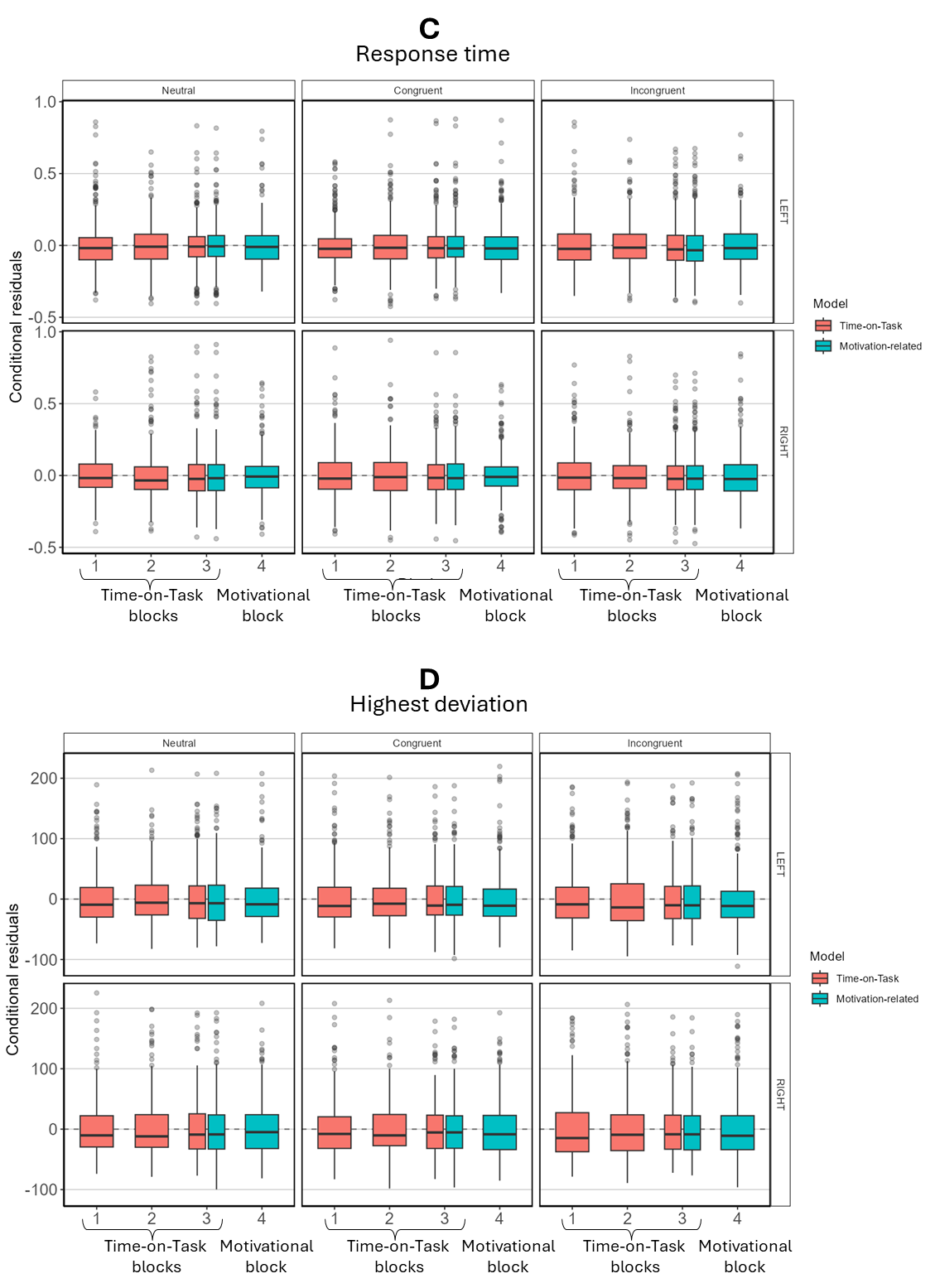


**Figure S18.** Conditional residual plots from LMM analyses for four performance measures (A–D) in Experiment 1.

**
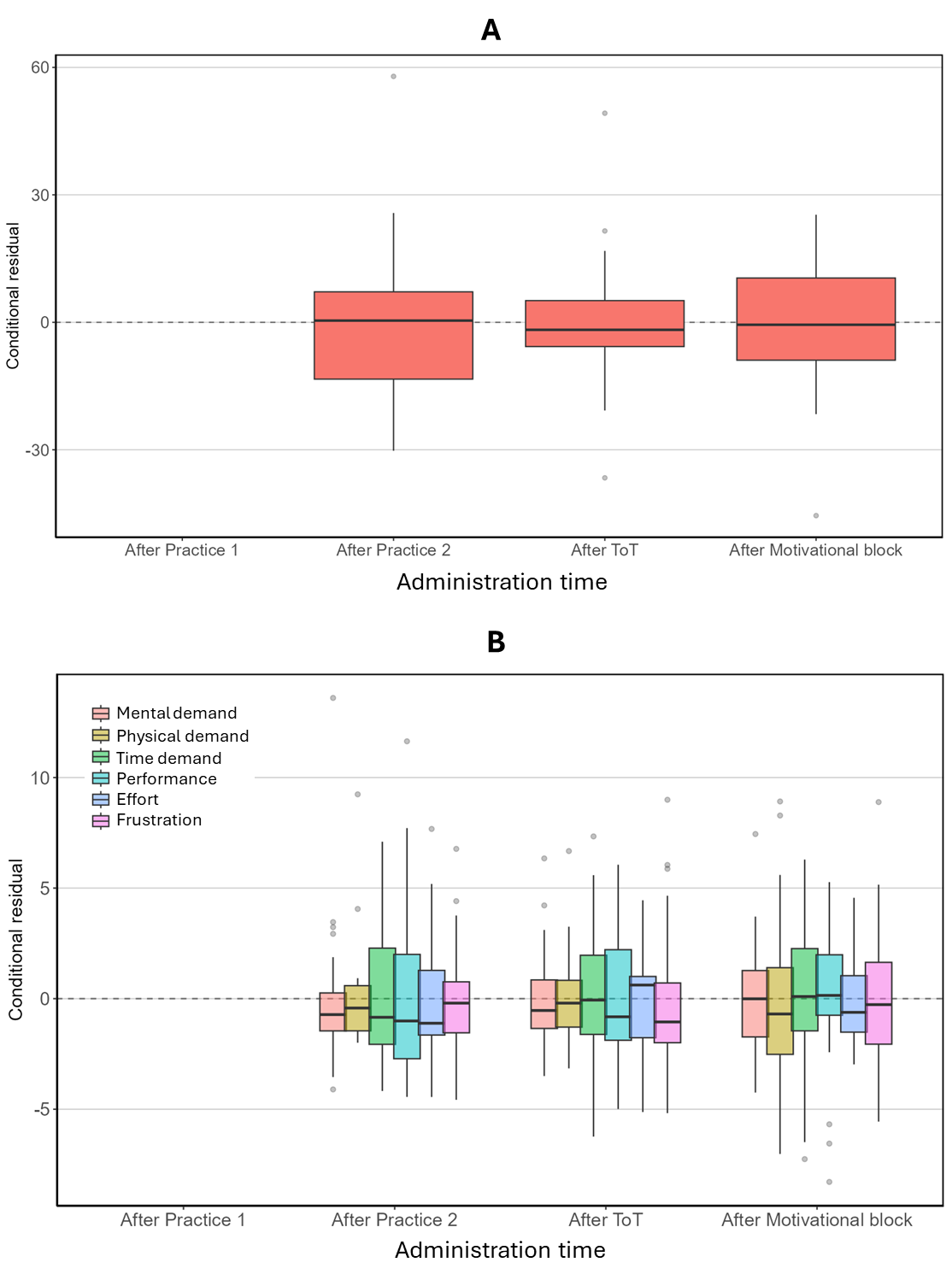
**

**Figure S19.** Conditional residual plots from LMM analyses for subjective fatigue (A) and perceived workload (B) in Experiment 2.


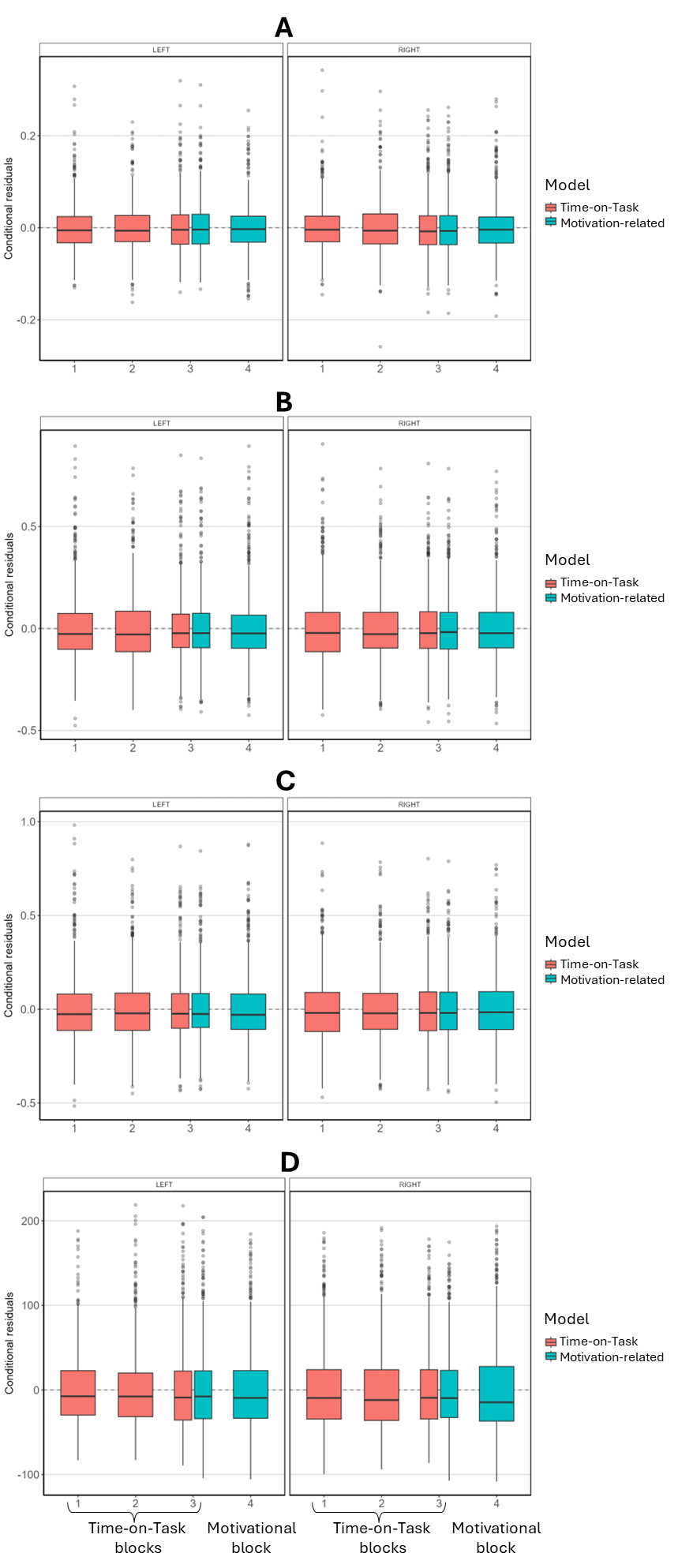


**Figure S20.** Conditional residual plots from LMM analyses addressed to the difference between the two target *presentation sides* (i.e. left vs. right target) for four performance measures (A: initiation time, B: movement time, C: response time, D: highest deviation) in Experiment 2.

**
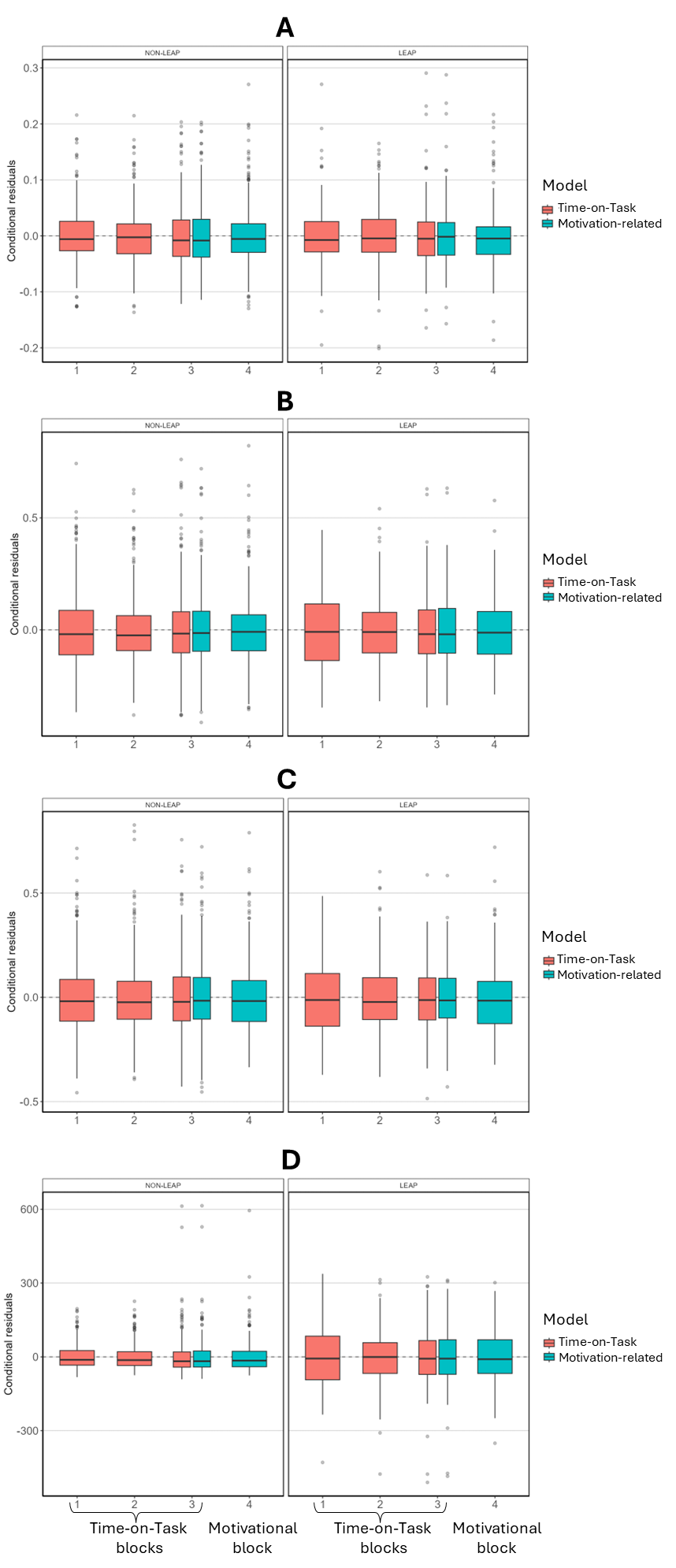
**

**Figure S21.** Conditional residual plots from linear mixed-effects model addressed to the difference between the *leap and normal trials* for four performance measures (A: initiation time, B: movement time, C: response time, D: highest deviation) in Experiment 2.

**Table S1.** Model estimates from linear mixed models in Experiment 1 (Time-on-Task phase).

| **Fixed effects** | **Performance metrics** | | | | | | | |
| --- | --- | --- | --- | --- | --- | --- | --- | --- |
|  | ***IT*** | | ***MT*** | | ***RT*** | | ***HD*** | |
|  | *Estimate* | *p* | *Estimate* | *p* | *Estimate* | *p* | *Estimate* | *p* |
| Intercept | .4482 | <.001 | .5620 | <.001 | 1.0099 | <.001 | 76.4978 | <.001 |
| Block1 | -.0078 | .0071 | -.0096 | .0603 | -.0166 | .0157 | -.5572 | .6217 |
| Block2 | -.0016 | .4502 | .0016 | .6146 | -.0002 | .9676 | 1.0454 | .4419 |
| Congruency1 | -.0095 | <.001 | -.0043 | .1334 | -.0135 | .0000 | -1.6385 | .0486 |
| Congruency2 | -.0104 | <.001 | -.0061 | .0349 | -.0164 | .0000 | -1.8964 | .0226 |
| Side1 | .0025 | .0072 | -.0085 | .0028 | -.0058 | .0629 | -3.2749 | .0117 |
| Block1:Congruency1 | .0002 | .8793 | -.0013 | .7487 | -.0007 | .8750 | -.2809 | .8107 |
| Block2:Congruency1 | .0014 | .2384 | .0011 | .7881 | .0020 | .6484 | -1.1585 | .3261 |
| Block1:Congruency2 | -.0017 | .1433 | -.0014 | .7383 | -.0029 | .4966 | -.5140 | .6611 |
| Block2:Congruency2 | -.0005 | .6925 | .0085 | .0377 | .0082 | .0545 | -.5068 | .6673 |
| Block1:Side1 | -.0022 | .0087 | -.0021 | .4719 | -.0041 | .1776 | .1133 | .8915 |
| Block2:Side1 | .0003 | .7596 | .0011 | .6938 | .0012 | .6962 | .0366 | .9651 |
| Congruency1:Side1 | -.0004 | .6046 | -.0022 | .4494 | -.0023 | .4469 | -.2633 | .7513 |
| Congruency2:Side1 | .0016 | .0586 | -.0013 | .6560 | -.0002 | .9456 | -.1870 | .8221 |
| Block1:Congruency1: Side1 | -.0000 | .9771 | .0087 | .0336 | .0094 | .0272 | -.1773 | .8798 |
| Block2:Congruency1: Side1 | .0020 | .0919 | -.0011 | .7850 | .0011 | .7984 | -.5806 | .6226 |
| Block1:Congruency2: Side1 | .0021 | .0684 | -.0007 | .8677 | .0009 | .8380 | .6072 | .6045 |
| Block2:Congruency2: Side1 | -.0011 | .3583 | .0043 | .2892 | .0038 | .3768 | -.3610 | .7594 |

**Table S2.** Model estimates from linear mixed models in Experiment 1 (Motivational phase).

| **Fixed effects** | **Performance metrics** | | | | | | | |
| --- | --- | --- | --- | --- | --- | --- | --- | --- |
|  | ***IT*** |  | ***MT*** |  | ***RT*** |  | ***HD*** |  |
|  | *Estimate* | *p* | *Estimate* | *p* | *Estimate* | *p* | *Estimate* | *p* |
| Intercept | .4495 | <.001 | .5613 | <.001 | 1.0110 | <.001 | 76.2994 | <.001 |
| Block3 | .0048 | .0465 | .0120 | .0195 | .0160 | .0126 | .4166 | .6920 |
| Congruency1 | -.0101 | .0000 | -.0071 | .1496 | -.0177 | .0008 | -.8230 | .5770 |
| Congruency2 | -.0090 | .0000 | -.0047 | .3495 | -.0130 | .0144 | -1.1668 | .4330 |
| Side1 | .0038 | .0006 | -.0120 | .0007 | -.0086 | .0220 | -4.3219 | .0000 |
| Block3:Congruency1 | -.0013 | .3815 | .0075 | .1277 | .0065 | .2164 | 1.0701 | .4680 |
| Block3:Congruency2 | .0021 | .1702 | -.0070 | .1592 | -.0063 | .2368 | -.2802 | .8510 |
| Block3:Side1 | .0007 | .4947 | .0007 | .8332 | .0012 | .7484 | -.0213 | .9840 |
| Congruency1:Side1 | -.0013 | .3890 | -.0028 | .5723 | -.0050 | .3393 | 1.1660 | .4290 |
| Congruency2:Side1 | .0031 | .0491 | .0000 | .9943 | .0039 | .4574 | -.8067 | .5890 |
| Block3:Congruency1:Side1 | -.0012 | .4259 | -.0055 | .2627 | -.0058 | .2658 | .5484 | .7100 |
| Block3:Congruency2:Side1 | .0005 | .7363 | -.0022 | .6662 | -.0030 | .5718 | -.3663 | .8060 |

**Table S3.** Model estimates from linear mixed models testing the effects of time-on-task, motivation and trial type (normal vs. leap trials) in Experiment 2.

| **Fixed effects** | **Performance metrics** | | | | | | | |
| --- | --- | --- | --- | --- | --- | --- | --- | --- |
|  | ***IT*** |  | ***MT*** |  | ***RT*** |  | ***HD*** |  |
|  | *Estimate* | *p* | *Estimate* | *p* | *Estimate* | *p* | *Estimate* | *p* |
| *Time-on-Task* |  |  |  |  |  |  |  |  |
| Intercept | .4609 | <.001 | .7331 | <.001 | 1.2022 | <.001 | 389.710 | <.001 |
| Block1 | -.0107 | .0155 | -.0043 | .4783 | -.0172 | .0088 | 2.9158 | .3497 |
| Block2 | .0007 | .8055 | -.0083 | .1780 | -.0026 | .6983 | -2.9550 | .3460 |
| Trial type1 | .0000 | .9997 | -.1255 | <.001 | -.1322 | <.001 | -303.903 | <.001 |
| Block1:Trial type1 | -.0007 | .7217 | -.0055 | .3703 | -.0045 | .4935 | -5.5903 | .0731 |
| Block2:Trial type1 | -.0027 | .1674 | -.0102 | .0988 | -.0161 | .0151 | .3833 | .9027 |
|  |  |  |  |  |  |  |  |  |
| *Motivation* |  |  |  |  |  |  |  |  |
| Intercept | .4625 | <.001 | .7316 | <.001 | 1.2002 | <.001 | 390.329 | <.001 |
| Block3 | .0082 | .0083 | .0121 | .0585 | .0188 | .0273 | -.1525 | .9620 |
| Trial type1 | .0016 | .3774 | -.1186 | <.001 | -.1239 | <.001 | -302.117 | <.001 |
| Block3:Trial type1 | .0018 | .3111 | .0088 | .0927 | .0116 | .0375 | 2.9827 | .2930 |

**Table S4.** Model estimates from linear mixed models testing the effects of time-on-task, motivation and side (left vs. right) in Experiment 2

| **Fixed effects** | **Performance metrics** | | | | | | | |
| --- | --- | --- | --- | --- | --- | --- | --- | --- |
|  | ***IT*** |  | ***MT*** |  | ***RT*** |  | ***HD*** |  |
|  | *Estimate* | *p* | *Estimate* | *p* | *Estimate* | *p* | *Estimate* | *p* |
| *Time-on-Task* |  |  |  |  |  |  |  |  |
| Intercept | .4639 | <.001 | .6092 | <.001 | 1.0734 | <.001 | 82.3025 | <.001 |
| Block1 | -.0116 | .0006 | -.0084 | .1842 | -.0201 | .0124 | -.3312 | .7350 |
| Block2 | .0002 | .9371 | -.0012 | .7593 | -.0010 | .8311 | -.1821 | .8530 |
| Side1 | -.0002 | .8678 | -.0128 | .0010 | -.0118 | .0014 | -4.4110 | <.001 |
| Block1:Side1 | -.0020 | .0779 | .0008 | .8303 | .0000 | .9925 | -.5938 | .5440 |
| Block2:Side1 | .0016 | .1619 | -.0003 | .9322 | .0009 | .8016 | -1.4169 | .1490 |
|  |  |  |  |  |  |  |  |  |
| *Motivation* |  |  |  |  |  |  |  |  |
| Intercept | .4665 | <.001 | .6020 | <.001 | 1.0687 | <.001 | 83.0590 | <.001 |
| Block3 | .0088 | .0017 | .0168 | .0008 | .0257 | .0002 | -.3351 | .7018 |
| Side1 | .0005 | .7181 | -.0102 | .0057 | -.0095 | .0030 | -4.0059 | <.001 |
| Block3:Side1 | -.0003 | .8303 | -.0028 | .3474 | -.0031 | .3338 | 1.7442 | .0464 |

**Table S5.** List of formulas used in linear mixed models in both experiments.

| **Experiment** | **Phase and analysis** | **Formula** |
| --- | --- | --- |
| EXP1 | Time-on-Task | Initiation time ~ Block*Congruency *Side+ (Block+Side\|Subject) |
| EXP1 | Time-on-Task | Movement time ~ Block*Congruency *Side+(Block+Side\|Subject) |
| EXP1 | Time-on-Task | Response time ~ Block*Congruency *Side+(Block+Side\|Subject) |
| EXP1 | Time-on-Task | Highest deviation ~ Block*Congruency *Side+(Block+Side\|Subject) |
| EXP1 | Motivation | Initiation time ~ Block*Congruency *Side+ (Block\|Subject) |
| EXP1 | Motivation | Movement time ~ Block*Congruency *Side+(Block\|Subject) |
| EXP1 | Motivation | Response time ~ Block*Congruency *Side+(Block\|Subject) |
| EXP1 | Motivation | Highest deviation ~ Block*Congruency *Side+(1\|Subject) |
| EXP2 | Time-on-Task, Trial type | Initiation time ~ Block*Trial type + (Block\|Subject) |
| EXP2 | Time-on-Task, Trial type | Movement time ~ Block*Trial type+(Trial type\|Subject) |
| EXP2 | Time-on-Task, Trial type | Response time ~ Block*Trial type+(Trial type\|Subject) |
| EXP2 | Time-on-Task, Trial type | Highest deviation ~ Block*Trial type+(Trial type\|Subject) |
| EXP2 | Time-on-Task, Side | Initiation time ~ Block * Side + (Block + Side \| Subject) |
| EXP2 | Time-on-Task, Side | Movement time ~ Block * Side + (Block + Side \| Subject) |
| EXP2 | Time-on-Task, Side | Response time ~ Block * Side + (Block + Side \| Subject) |
| EXP2 | Time-on-Task, Side | Highest deviation ~ Block*Side+(1\|Subject) |
| EXP2 | Motivation, Trial type | Initiation time ~ Block*Trial type + (Block\|Subject) |
| EXP2 | Motivation, Trial type | Movement time ~ Block * Trial type + (Block \| Subject) |
| EXP2 | Motivation, Trial type | Response time ~ Block * Trial type + (Block \| Subject) |
| EXP2 | Motivation, Trial type | Highest deviation ~ Block*Trial type+(Block+Trial type\|Subject) |
| EXP2 | Motivation, Side | Initiation time ~ Block * Side + (Block* Side \| Subject) |
| EXP2 | Motivation, Side | Movement time ~ Block * Side + (Block+ Side \| Subject) |
| EXP2 | Motivation, Side | Response time ~ Block * Side + (Block \| Subject) |
| EXP2 | Motivation, Side | Highest deviation ~ Block*Side+(1\|Subject) |

| **Random effects correlation** | **Performance metrics** | | | |
| --- | --- | --- | --- | --- |
|  | **Initiation time** | **Movement time** | **Response time** | **Highest deviation** |
| *Time-on-Task* |  |  |  |  |
| Block1–Block2 | 0.16 | 0.30 | 0.33 | -0.14 |
| Block1–Block3 | -0.02 | 0.34 | 0.28 | -0.38 |
| Block1–Side (right) | -0.08 | -0.25 | -0.38 | -0.66 |
| Block2–Block3 | 0.78 | 0.91 | 0.81 | 0.61 |
| Block2– Side (right) | -0.26 | 0.44 | -0.05 | 0.25 |
| Block3– Side (right) | -0.10 | 0.21 | 0.00 | 0.77 |
| *Motivation* |  |  |  |  |
| Block3-Block4 | -0.64 | -0.82 | -0.86 | NA |

**Table S6.** Correlations between random effects in the LMMs in Experiment 1.

*Note:* Block1 refers to individual differences in the first block of trials. Block2 and Block3 indicate changes from Block1 to Block2 and Block3, respectively. In the motivation-related analyses, Block3 refers to individual differences in Block3, while Block4 indicates the change from Block3 to the motivational block. NA = not applicable; the model did not include the corresponding random effect.

| **Random effects correlation** | **Performance metrics** | | | |
| --- | --- | --- | --- | --- |
|  | **Initiation time** | **Movement time** | **Response time** | **Highest deviation** |
| *Time-on-Task* |  |  |  |  |
| Block1–Block2 | 0.06 | 0.30 | 0.36 | NA |
| Block1–Block3 | 0.08 | 0.04 | 0.17 | NA |
| Block1–Side (right) | 0.26 | 0.00 | -0.07 | NA |
| Block2–Block3 | 0.74 | 0.90 | 0.91 | NA |
| Block2– Side (right) | -0.03 | 0.06 | -0.35 | NA |
| Block3– Side (right) | -0.35 | 0.21 | -0.09 | NA |
| *Motivation* |  |  |  |  |
| Block3-Block4 | -0.3 | -0.58 | -0.5 | NA |
| Block3–Side (right) | -0.01 | 0.36 | NA | NA |
| Block3–Block4×Side (right) | -0.72 | NA | NA | NA |
| Block4–Side (right) | 0 | -0.68 | NA | NA |
| Block4–Block4×Side (right) | -0.12 | NA | NA | NA |
| Side (right)–Block4×Side (right) | -0.06 | NA | NA | NA |

**Table S7.** Correlations between random effects in the LLMs addressed to the difference between the two target presentation sides (i.e. left vs. right target) in Experiment 2.

*Note:* Block1 refers to individual differences in the first block of trials. Block2 and Block3 indicate changes from Block1 to Block2 and Block3, respectively. In the motivation-related analyses, Block3 refers to individual differences in Block3, while Block4 indicates the change from Block3 to the motivational block. NA = not applicable; the model did not include the corresponding random effect.

| **Random effects correlation** | **Performance metrics** | | | |
| --- | --- | --- | --- | --- |
|  | **Initiation time** | **Movement time** | **Response time** | **Highest deviation** |
| *Time-on-Task* |  |  |  |  |
| Block1–Block2 | -0.08 | NA | NA | NA |
| Block1–Block3 | -0.21 | NA | NA | NA |
| Block1–Trial type (leap) | NA | 0.55 | -0.47 | -0.12 |
| Block2–Block3 | 0.82 | NA | NA | NA |
| *Motivation* |  |  |  |  |
| Block3-Block4 | -0.23 | -0.46 | -0.42 | -0.88 |
| Block3–Trial type (leap) | NA | NA | NA | -0.05 |
| Block4-Trial type (leap) | NA | NA | NA | 0.26 |

**Table S8**. Correlations between random effects in the LMMs addressed to the difference between the leap and normal trials in Experiment 2.

*Note:* Block1 refers to individual differences in the first block of trials. Block2 and Block3 indicate changes from Block1 to Block2 and Block3, respectively. In the motivation-related analyses, Block3 refers to individual differences in Block3, while Block4 indicates the change from Block3 to the motivational block. NA = not applicable; the model did not include the corresponding random effect.
